# Optimization of Segmented Thermoelectric Generator with Wavy Structure and Nanofluid Cooling: Variable Structure

**DOI:** 10.3390/nano16191248

**Published:** 2026-10-02

**Authors:** Edgar Alan De Los Santos-Castañeda, Sara Eunice Olivares-Hernández, Miguel Ángel Olivares-Robles, Pedro Guevara-López

**Affiliations:** 1 SEPI ESIME Culhuacán, Instituto Politécnico Nacional, Coyoacán, Ciudad de México 04430, Mexico; edelossantosc1701@alumno.ipn.mx (E.A.D.L.S.-C.); pguevara@ipn.mx (P.G.-L.); 2 Facultad de Ciencias, UNAM, Investigación Científica, Ciudad Universitaria, Coyoacán, Ciudad de México 04510, Mexico; saraoliv@ciencias.unam.mx

**Keywords:** thermoelectric generator, segmented thermoelectric legs, sinusoidal geometry, nanofluid, voltage

## Abstract

Thermoelectric generators (TEGs) are highly sensitive to both geometric configuration and thermal boundary conditions, particularly the heat dissipation mechanisms at the cold side. Despite extensive research on thermoelectric materials, cooling strategies, and geometric optimization, the coupled influence of hollow sinusoidal geometries and convective cooling conditions remains insufficiently understood. Therefore, this work presents an integrated electrothermal optimization of a segmented hollow sinusoidal thermoelectric generator using a finite element model developed in ANSYS Workbench 2023 R1. The proposed TEG is composed of hollow thermoelements segmented with Bi_2_Te_3_ and skutterudite using a fixed segmentation ratio. Attention is given to the effects of the revolution distance ($d_{r}$) and inter-leg spacing (D), geometric parameters that have received limited attention in previous studies. The performance of the device was evaluated by varying the revolution distance from 0.15 mm to 0.58 mm under applied heat fluxes ($q^{\prime \prime }$) ranging from 5000 to 35,000 W/$m^{2}$. Additionally, the influence of inter-leg spacing on voltage generation was systematically investigated. Cooling conditions included both an idealized constant-temperature boundary condition of 25 °C, employed for model validation, and convective cooling conditions based on ${TiO}_{2}$, graphene, distilled water, ${Fe}_{3}O_{4}$, and ${Al}_{2}O_{3}$ nanofluids. The convective heat transfer coefficients were determined from nanofluid thermoelectric properties and implemented as thermal boundary conditions at the cold side of the thermoelectric generator. Among the cooling media evaluated, ${TiO}_{2}$-based cooling exhibited the closest performance to constant-temperature condition. Nevertheless, the assumption of a constant cold-side temperature was found to overestimate the thermoelectric performance compared with convective cooling conditions scenarios. The results revealed that both geometric parameters and cooling conditions significantly affect the electrothermal behavior of the device. The maximum output voltage of approximately 0.39 V was obtained for $d_{r}=0.15$ mm under ${TiO}_{2}$-based cooling and an applied heat flux of 35,000 W/$m^{2}$. Increasing the revolution distance from 0.15 mm to 0.58 mm reduced voltage generation. Furthermore, the inter-leg spacing exhibited a non-linear influence on voltage generation, highlighting the complex interaction between geometry and thermal transport mechanisms. The findings also demonstrate the importance of simultaneously considering revolution distance, inter-leg spacing, thermal loading, and convective nanofluid-based cooling conditions during thermoelectric generator design and optimization.

## 1. Introduction

The continuous growth in global energy demand has led to a substantial increase in greenhouse gas emissions associated with electricity generation, heating, cooling, and air-conditioning systems. Despite significant advancements in renewable and alternative energy technologies, current energy production and utilization processes still involve considerable environmental impacts and intensive resource consumption [1,2]. Moreover, the increasing energy demand indicates that the challenge is not limited to energy sources, but also to the inefficient use of available thermal resources. In this context, improving energy efficiency and recovering waste heat have become key strategies for mitigating environmental and energy-related challenges [3,4].

Thermoelectric technology has attracted considerable attention because it enables the direct conversion of thermal energy into electricity without the need for moving components. This characteristic makes thermoelectric generators attractive for applications in waste-heat recovery, remote power generation, and energy harvesting [5,6,7]. Due to the absence of moving parts, thermoelectric generators (TEGs) offer silent operation, high reliability, low maintenance requirements, and excellent scalability over a wide range of power outputs. One of the earliest practical applications of TEG was implemented in the U.S. Navy Transit satellite in 1961, where Pb-Te-based materials were used to generate approximately 2.7 W of electrical power under space conditions [8].

In industrial applications, thermoelectric Technology has been successfully employed for waste heat recovery, particularly in systems developed by companies such as Gentherm (formerly Global Thermoelectric). These systems utilize heat released from fuel combustion (e.g., natural gas, butane, or propane). In such configurations, a burner heats one side of thermoelectric modules, while the opposite side is cooled by fin-based heat exchangers that promote natural convection. Depending on the design and operating conditions, these systems can typically generate between 15 and 550 W of electrical power [9].

In the automotive sector, thermoelectric Technology has also demonstrated potential for improving energy efficiency through exhaust heat recovery. For example, General Motors integrated a TEG system into a Chevrolet Suburban, using ${Bi}_{2}{Te}_{3}$-based modules installed along the exhaust line [5,6]. A bypass mechanism was implemented to regulate the module temperature and prevent it from exceeding 250 °C, despite exhaust gases reaching approximately 400 °C. Under these operating conditions, the system achieved a power output close to 25 W, highlighting both the potential and current limitations of thermoelectric materials in vehicular applications [10,11].

Recent developments in thermoelectric materials have focused on increasing the figure of merit through nano structuring [12], band engineering, and segmentation approaches. These strategies have improved energy conversion efficiency and expanded the range of practical applications of thermoelectric generators. These developments reinforce the growing relevance of thermoelectric Technology from both technological and commercial perspectives, particularly in the context of sustainable energy systems and waste heat recovery applications [13].

Recently, geometric optimization has emerged as an effective approach for enhancing heat-transfer performance in energy systems. Chunyan Zhao et al. [14] demonstrated that variable-density pin-fin arrangements significantly improve heat dissipation while minimizing pressure-drop penalties, whereas Hongxin Deng et al. [15] reported that aircraft-shaped varied-section ribs enhance convective heat transfer through flow recirculation and thermal boundary-layer disruption. These studies highlight the strong influence of geometric modifications on heat-transfer mechanisms and thermal transport. Motivated by these findings, the present work investigates how the geometric parameters of hollow sinusoidal thermoelements modify the heat-transfer pathway, temperature gradients, and voltage generation in a segmented thermoelectric generator.

Several studies have shown that modifying the geometry of thermoelectric legs can significantly improve performance. For instance, triangular leg configurations, as reported by Li et al. [16], can generate up to 100% greater power output compared to conventional rectangular geometries under heat flux conditions ranging from $4000\;to\;16,000\;W/m^{2}.$ This improvement is primarily attributed to the reduction in internal electrical resistance, quantified by the parameter $R_{A}$, defined as the ratio between the hollow area and the total cross-sectional area. This parameter reduces material usage while enabling higher current flow, thereby increasing power output.

The influence of thermoelectric leg geometry has also been explored through alternative configurations such as rectangular, trapezoidal, and hollow structures [17,18,19,20]. These studies demonstrate that while rectangular legs tend to maximize power output, non-uniform and hollow geometries improve efficiency by reducing internal electrical resistance and material usage. Additionally, optimal leg lengths in the range of 2–4 mm have been identified to balance thermal gradients and electrical resistance, providing a basis for efficient thermoelectric design [21].

${Bi}_{2}{Te}_{3}$ is widely recognized as the benchmark material for low-temperature thermoelectric applications, with optimal performance near 300 K, where it achieves figure-of-merit values close to ZT≈1. Its alloys, such as Bi_2−x_Sb_x_Te_3_ and Bi_2_Te_3−x_Se_x_, can improve thermoelectric performance through doping and defect engineering, thereby enhancing the Seebeck coefficient and charge-carrier transport. As a result, ${Bi}_{2}{Te}_{3}$ remains the dominant material in commercial thermoelectric devices operating under low to moderate temperature gradients [22].

In contrast, skutterudite-based materials exhibit superior performance in intermediate temperature ranges (200–700 °C). Their cage-like crystal structure allows the incorporation of filler atoms, which enhances phonon scattering and significantly reduces lattice thermal conductivity while maintaining favorable electrical properties. This combination enables higher ZT values at elevated temperatures, making skutterudites particularly suitable for waste heat recovery in industrial and automotive applications [23].

Due to the strong temperature dependence of thermoelectric material properties, a single material is typically unable to maintain optimal performance across a wide temperature range. To address this limitation, segmented thermoelectric generators (STEGs) combine multiple materials along the leg length, allowing each segment to operate within its optimal temperature range [24,25,26].

Several studies have demonstrated the effectiveness of segmentation strategies. For example, Ali and Yilbas [27] showed that combining PbTe in the hot region with ${Bi}_{2}{Te}_{3}$ in the cold region improves conversion efficiency by up to 8% compared to non-segmented designs. Similarly, Shen et al. [28] reported that optimized segment distributions can significantly enhance cooling capacity and coefficient of performance. Other works have confirmed that the optimal segmentation ratio depends on the operating temperature and material distribution, requiring a balance between efficiency, power output, and mechanical stability [29,30]. These findings demonstrate that segmentation is a key strategy for improving thermoelectric performance by better matching material properties to the temperature gradient within the device [31].

In addition to material optimization, geometric modification of thermoelectric legs has emerged as an effective strategy to enhance device performance. Among these approaches, wavy-shaped and hollow geometries have received increasing attention due to their ability to extend the effective thermal and electrical transport paths without increasing the overall device dimensions [32].

Ali and Moria [33] proposed a novel thermoelectric generator design featuring hollow sinusoidal legs, where a sinusoidal profile defines the geometry. This configuration increases the effective conduction length and promotes a more favorable temperature distribution along the thermoelectric elements, resulting in improved voltage generation. However, their study assumed an idealized constant cold-side temperature of 25 °C, which limits the applicability of the results under convective operating conditions.

One of the main limitations found in previous thermoelectric studies is the frequent assumption of a constant cold-side temperature. Although this approach simplifies numerical modeling and facilitates comparisons between different configurations, it does not adequately represent the thermal conditions encountered in practical applications. For this reason, several researchers have investigated alternative cooling strategies to improve heat extraction from the cold side of the thermoelectric module. Experimental and numerical studies have demonstrated that nanofluids such as $TiO_{2}$, ${Al}_{2}O_{3}$, and graphene can enhance heat transfer performance compared to conventional fluids, leading to improved thermoelectric efficiency and power output [34,35]. These improvements are primarily attributed to mechanisms such as Brownian motion and enhanced thermal transport at the fluid–solid interface [36].

Despite these advances, most existing studies evaluate thermoelectric performance under simplified thermal boundary conditions, such as constant cold-side temperature assumptions, which may lead to an overestimation of the real device performance. Furthermore, the combined effect of segmented materials, complex leg geometries, and convective cooling has not been fully explored.

Although previous studies have investigated segment thermoelectric generators, hollow or wavy leg geometries, and nanofluid-based cooling independently, the combined influence of these design strategies has not been systematically evaluated. No previous study has analyzed a segment $Bi_{2}Te_{3}$–skutterudite thermoelectric generator with hollow sinusoidal legs under nanofluid-convection boundary conditions while simultaneously assessing the effects of revolution distance ($d_{r}$) and inter-leg spacing (D).

Therefore, the present study addresses this research gap through a three-dimensional finite-element model of a segmented Bi_2_Te_3_/skutterudite thermoelectric generator with hollow sinusoidal thermoelements. Temperature-dependent material properties and convective cooling boundary conditions based on different nanofluids are considered. Particular attention is given to the effects of sinusoidal cycle number, revolution distance $d_{r}$, and inter-thermoelement spacing D on temperature distribution, voltage generation, power output, and conversion efficiency.

## 2. Materials and Methods

In the present study, a thermoelectric generator (TEG) composed of two segmented thermocouples is modeled numerically. Each thermoelectric leg features an axially segmented structure: the lower segment of the thermocouple, which is in direct contact with the heat source, is made of skutterudite (both n−type and p−type), while the upper segment, adjacent to the heat sink, is composed of bismuth telluride (${Bi}_{2}{Te}_{3}$). From a geometric perspective, the TEG configuration is constructed from a wavy-leg profile defined by Equation (1) [33],

(1) $$Y=A_{w}\sin\left(\frac{2N\pi S}{h_{l}}\right)$$

Here, S represents the axial coordinate along the leg length $\left(0\leq S\leq h_{l}\right)$, $A_{w}$ denotes the wave amplitude, N is the number of sinusoidal cycles, and $h_{l}$ corresponds to the total leg height, which is fixed at 2 mm for each thermoelectric leg. Based on this two-dimensional profile, the three-dimensional geometry of each leg is generated using a solid-of-revolution operation.

A distance radially offsets the axis of revolution $d_{r}$ with respect to the sinusoidal profile described by Equation (1), creating a controlled separation between the axis of revolution and the geometric profile, as shown in Figure 1a. The magnitude of $d_{r}$ determines the size of the internal cavity and enables the formation of a hollow structure with a uniform wall thickness, as illustrated in Figure 1b. The base geometry used in this study was constructed following the dimensions proposed by Ali and Moria [33]. The parametric analysis focuses exclusively on variations in the radial offset $d_{r}$ and the spacing distance between thermoelectric elements D, to evaluate the combined influence of these parameters on the thermoelectric performance of the generator.

The selection of a hollow-leg configuration is based on fundamental geometric and functional considerations. This design allows the effective implementation of an undulated profile on the external surface of the thermoelectric leg, increasing the effective thermal and electrical conduction length without requiring a significant increase in cross-sectional area.

### 2.1. Selection of Geometrical Parameter Ranges

The ranges investigated of the revolution distance ($d_{r}$) and spacing between adjacent thermoelements (D) were defined according to the geometric constraints of the proposed thermoelectric architecture. The lower bound of the revolution distance, $d_{r,min}=0.15$ mm, corresponds to the minimum admissible distance between the sinusoidal profile and the axis of revolution while preserving the intended hollow geometry of the thermoelement. Conversely, the upper bound, $d_{r,max}=0.58$ mm, ensures constant alumina cover dimensions throughout the thermoelectric module. Larger values would increase the thermoelement cross-sectional area and modify the available external surface area, leading to deviations from the intended module configuration. It is important to note that variations in $d_{r}$ do not modify the overall thermoelement height, wall thickness, or the effective sinusoidal conduction pathway. Instead, changing $d_{r}$ only alters the size of the internal cavity and, consequently, the effective cross-sectional area available for heat and electrical transport. Therefore, the thermoelement thickness remains constant throughout all investigated configurations, and the observed thermoelectric variations are primarily associated with changes in the internal cavity geometry rather than modifications of the conduction path. The influence of the revolution distance on the thermoelectric response of the proposed design is subsequently analyzed and discussed in Section 3.3.

Similarly, the spacing between adjacent thermoelements varied from 0.05 mm to 0.85 mm. The lower limit was selected to avoid geometric interference between neighboring thermoelements, whereas the upper limit preserves the overall dimensions of the thermoelectric module while maintaining the proposed geometric configuration. Previous studies have identified thermoelement spacing as an important geometric parameter due to its influence on heat transfer and on the overall thermoelectric response of the module. For instance, Abd El-Moneim A. Harb and co-workers [37], investigated n/p couple spacings ranging from 0.25 mm to 1.5 mm and demonstrated that thermoelement separation significantly affects the thermal and electrical behavior of thermoelectric generators. Consequently, thermoelement spacing is considered a relevant design variable in the present work. However, unlike the configuration studied by Abd El-Moneim A. Harb et al. [37], the spacing range considered here is additionally constrained by the geometric limits of the proposed hollow architecture and by the requirement of preserving the module dimensions.

### 2.2. Physical Model

In the case of a solid leg, the introduction of external undulations does not result in a substantial increase in the effective conduction pathway due to the presence of a solid internal core with limited thermal and electrical participation. The hollow geometry, however, enables the sinusoidal shape to be fully exploited, extending both thermal and electrical transport paths thereby modifying the thermal and electrical transport characteristics and the resulting Seebeck voltage [33]. Furthermore, the hollow structure provides a more general and flexible geometrical model, capable of representing a wide variety of configurations and cross-sectional shapes. This versatility facilitates the extension of the proposed methodology to other geometries commonly employed in commercial or experimental thermoelectric generators [16].

The proposed thermoelectric generator (TEG) model consists of two semiconductor elements: one p−type and one n−type. Both elements feature a segmented configuration, employing skutterudite in the lower section and ${Bi}_{2}{Te}_{3}$ in the upper section. The elements are electrically connected in series through copper interconnectors with a thickness of $h_{i}=0.25\;mm.$ The junction between the copper and the thermoelectric elements is achieved by means of solder layers with a thickness of $h_{s}=0.125\;mm$, while ceramic layers provide electrical insulation with a thickness of $h_{c}=0.5\;mm$.

The thermoelectric elements have a hollow geometry with a uniform wall thickness along their entire length, ensuring electrical continuity and adequate thermal contact. Figure 2 presents a detailed view of the computational domain, highlighting the main geometric parameters considered in the model. The height of the thermoelectric legs is set to $h_{l}=2.0\;mm$, with a revolution distance of $d_{r}=0.28\;mm$ and a spacing between adjacent legs of D=0.5 mm [6,14].

### 2.3. Material Properties

Thermoelectric materials generally exhibit a strong dependence of their performance on operating temperature, which represents a major limitation for achieving high efficiency over wide temperature gradients. In the proposed TEG, the legs are composed of both n−type and p−type thermoelectric materials, bismuth telluride and skutterudite—whose transport properties are strongly temperature-dependent. As shown in Figure 3, electrical resistivity, Seebeck coefficient, and thermal conductivity vary significantly with temperature for both n−type and p−type materials, directly influencing device performance. Most thermoelectric materials are optimized to operate within a relatively narrow temperature range and experience a significant reduction in efficiency outside their optimal working conditions.

Segmented thermoelectric generators provide an effective strategy to overcome this limitation by combining materials optimized for different temperature ranges within the same device. In this configuration, bismuth telluride (${Bi}_{2}{Te}_{3}$), which exhibits optimal performance around 250 °C, is positioned in the low-temperature region, while skutterudite, with optimal performance at elevated temperatures (approximately 600 °C), is placed in the high-temperature region. This spatial arrangement allows the thermoelectric legs of the TEG to maintain high performance over a broader temperature gradient, enabling a more efficient utilization of the available thermal energy and enhancing the overall system efficiency [38]. Additional material properties considered in the numerical simulations, including thermal expansion coefficient, density, Young’s modulus, heat capacity, Poisson’s ratio, and electrical and thermal conductivities, are summarized in Table 1.

The geometry of the thermoelectric leg directly influences both the temperature gradient, and the electrical potential developed along its length. In hollow configurations with a sinusoidal profile, such as those considered in this study. In hollow sinusoidal configurations, geometric variations modify the effective cross-sectional area available for heat and charge transport, thereby affecting the temperature distribution and the resulting Seebeck voltage generated by the thermoelectric device.

The segmentation strategy was selected to exploit the temperature-dependent behavior of thermoelectric materials. Skutterudite exhibits superior thermoelectric performance at elevated temperatures, whereas bismuth telluride provides maximum efficiency near room temperature. Therefore, placing skutterudite in the hot region and $Bi_{2}Te_{3}$ in the cold region allows each material to operate closer to its optimal temperature range, improving the overall thermoelectric conversion performance and enabling a more efficient utilization of the available thermal gradient.

### 2.4. Governing Equations

The thermoelectric generator is modeled by solving the coupled thermal and electrical transport equations under steady-state conditions. The governing equations are derived from the conservation of energy and electric charge and account for the Seebeck, Peltier, Fourier, and Joule effects. The thermo-electrical coupling relates the temperature field and the electric potential through material-dependent properties such as thermal conductivity, electrical conductivity, and the Seebeck coefficient, following the formulation reported by Ramos-Castañeda et al. [34]. The heat flux, thermoelectric current, and generated voltage are expressed by Equations (2)–(4), respectively. The heat flux is given by

(2) $$\vec{q}=\Pi \;\vec{J}-k\nabla T$$

where $\vec{q}$ is the heat flux vector (W/$m^{2}$), Π is the Peltier coefficient (V), $\vec{J}$ is the electric current density vector (A/$m^{2})$, k is the thermal conductivity tensor (W/m·°C), and ∇T is the temperature gradient (°C/m).

The electric current density is described by [16]:

(3) $$\vec{J}=\sigma (\vec{E}-\alpha \nabla T)$$

where σ is the electrical conductivity (S/m), $\vec{E}$ is the electric field intensity (V/m), and α is the Seebeck coefficient (V/°C).

The electric field is related to the electric potential through:

(4) $$\vec{E}=-\nabla \phi$$

where ϕ represents the electric potential (V).

The thermoelectric coupling between the Seebeck and Peltier effects is established through Kelvin’s relation:

(5) Π=αT

where T is the absolute temperature (K).

The open-circuit voltage generated by the thermoelectric module is determined from the Seebeck relation [16]:

(6) $$V_{oc}=\alpha \Delta T$$

where $V_{oc}$ is the open circuit, α is, the Seebeck coefficient and ΔT is the temperature difference between the hot and cold sides.

According to the maximum power transfer conditions, the load resistance is assumed to be equal to the internal electrical resistance of the thermoelectric module [16]:

(7) $$R_{L}=R_{int}$$

where $R_{L}$ is the external load resistance and $R_{int}$ is the internal electrical resistance of the thermoelectric module.

Under the matched-load condition $R_{L}=R_{int}$, the current delivered to the load is:

(8) $$I=\frac{V_{oc}}{2R_{int}}$$

where I is the electrical current delivered to the load.

The maximum electrical power delivered to the load is calculated as:

(9) $$P_{max}=\frac{{V_{oc}}^{2}}{{4R}_{int}}$$

where $P_{max}$ is the maximum electrical output power, $V_{oc}$ is the open-circuit voltage, and $R_{int}$ is the internal electrical resistance.

The heat absorbed at the hot side of the thermoelectric module is expressed as:

(10) $$Q_{h}=\alpha T_{h}I-\frac{1}{2}I^{2}R_{int}+K\Delta T$$

where $Q_{h}$ is the heat absorbed at the hot side, $T_{h}$ is the hot-side temperature, I is the electrical current, and K is the thermal conductance of the thermoelectric module. The three terms represent the Peltier heat, Joule heating contribution, and conductive heat transfer, respectively.

Finally, under the maximum power condition $\left(R_{L}=R_{int}\right)$, the thermoelectric conversion efficiency is calculated as:

(11) $$\eta_{R_{L}=R_{int}}=\frac{P_{max}}{Q_{h}}$$

where $\eta_{R_{L}=R_{int}}$ is the thermoelectric conversion efficiency at the maximum power condition, $P_{max}$ is the maximum electrical output power, and $Q_{h}$ is the heat absorbed at the hot side.

The conservation of electric charge is expressed by

(12) ∇·J=0

While the conservation of energy is given by

(13) ∇·q+Q=0

where Q represents the volumetric heat generation associated with Joule heating and thermoelectric effects.

These equations are solved simultaneously to obtain the temperature distribution, electric potential. current density, and output voltage throughout the segmented thermoelectric generator.

### 2.5. Mesh Independence Study

The total heat flux within the thermoelectric elements is computed as the superposition of conductive heat transfer and thermoelectric transport. In the present study, the governing equations are discretized using the finite element method, resulting in the coupled system of algebraic equations given by Equation (10). In this framework, the voltage response gradient established across the thermoelectric legs under different geometric and cooling configurations is obtained [39,40].

(14) $$\left[\begin{array}{cc} K_{TT} & K_{T\phi }\\ K_{\phi T} & K_{\phi \phi } \end{array}\right]\left\{\begin{array}{c} T\\ \phi \end{array}\right\}=\left\{\begin{array}{c} F_{T}\\ F_{\phi } \end{array}\right\}$$

where $K_{TT}$ represents the thermal conductivity matrix, $K_{\phi \phi }$ represents the electrical conductivity matrix, and $K_{T\phi }$ and $K_{\phi T}$ denote the thermoelectric coupling matrices associated with Peltier and Seebeck phenomena, respectively. The vectors $F_{T}$ and $F_{\phi }$ contain the thermal and electrical load contributions.

The coupled thermal and electrical equations are solved using a thermoelectric module implemented in a Multiphysics simulation environment, which provides the temperature and electric potential distribution within the thermoelectric generator.

Figure 4 presents the computational mesh used in the thermoelectric generator, under the design conditions previously defined in Figure 1 and Figure 2. An unstructured tetrahedral mesh was generated to accurately capture the complex sinusoidal geometry of the hollow thermoelectric legs. Local mesh refinement was applied in regions with high geometric curvature and at material interfaces, including the thermoelectric legs, solder layers, intermediate layers, and copper interconnectors, to ensure an accurate resolution of thermal and electrical gradients. Additionally, coarser elements were employed in regions with more uniform geometry, such as ceramic substrates, to reduce the overall computational cost without compromising numerical accuracy. A cross-sectional view of the TEG is also shown in Figure 4, where the distribution of the tetrahedral mesh and the level of refinement achieved in critical regions of the model can be clearly observed.

To assess mesh independence of the computational model, a grid independence study was conducted under steady-state conditions. The analysis was performed for a representative case with the following parameters: $h_{r}=1\;mm,$ $A_{w}=0.05\;mm$, $t_{l}=0.2\;mm$, N=1, and $q^{\prime \prime }=20,000\;W/m^{2}$. The thermoelectric generator (TEG) performance was evaluated using a multiphysics numerical approach, and a mesh independence study was carried out to minimize the uncertainty associated with the simulations, following methodologies commonly reported in the literature, such as those by Aly and Moria et al. [33]. The voltage values obtained for different total numbers of elements are presented in Table 2.

To further quantify the numerical uncertainty associated with the mesh discretization, the relative error between consecutive mesh refinements was evaluated according to [39]:

(15) $$\epsilon =\frac{\left|V_{i}-V_{i-1}\right|}{V_{i}}\times 100$$

where $V_{i}$ and $V_{i-1}$ are the voltages obtained from the current and previous mesh, respectively. This metric was used to assess mesh convergence and verify that further refinement had a negligible influence on the numerical solution.

Figure 5 presents the mesh convergence analysis for the thermoelectric generator. As the number of mesh elements increases, the predicted output voltage approaches an asymptotic value, indicating numerical convergence. The largest variation is observed between the two course meshes, whereas subsequent refinements produce only minor changes in the predicted voltage. For meshes containing more than approximately 10,000 elements, the relative error remains below 0.1%, confirming that the solution becomes practically independent of mesh density.

Based on the mesh independence study, the mesh with 146,968 elements was selected because further mesh refinement produced negligible changes in the output voltage, indicating that the numerical solution had reached convergence.

### 2.6. Nanofluid System

The calculated coefficients represent the imposed convective cooling conditions at the upper cold-side ceramic surface. The contact area between the nanofluid and the solid surface is assumed to be equal to the substrate area of the TEG. The flow is considered laminar and hydrodynamically developing, while transient effects are neglected. Under these assumptions, the convective heat-transfer characteristics are evaluated using the Sieder–Tate correlation for the Nusselt number [23,24].

The Nusselt number is expressed as:

(16) Nu=1.86RePrlde1/3

where $\mathrm{Pr}=\rho \nu C_{p}/\kappa$ is the Prandtl number, Re = $\upsilon d_{e}/\nu$ is the Reynolds number, $d_{e}=4A_{f}/B$ is the equivalent hydraulic diameter, υ is the flow velocity, ν is the kinematic viscosity, $A_{f}$ is the flow cross-sectional area, and B is the wetted perimeter. The convective heat-transfer coefficient is then calculated as:

(17) $$h=\frac{\kappa Nu}{l}$$

The computed heat-transfer coefficient is applied as a boundary condition at the cold side of the thermoelectric generator for the nanofluid-cooled cases. Heat losses from the thermoelectric elements, as well as thermal resistances in the cooling channel and ceramic plates, are neglected to focus on the influence of convective cooling on thermoelectric performance.

The thermophysical properties of the base fluid, nanoparticles, and resulting nanofluids employed in the cooling system are summarized in Table 3. These properties are used to evaluate the Reynolds, Prandtl, and Nusselt numbers and subsequently determine the convective heat-transfer coefficient [8,10,11].

Using Equations (16) and (17), the convective heat-transfer coefficient was determined for each nanofluid under the selected operating condition (Re=2500). Since the nanofluid flows externally over the upper cold-side ceramic surface of the thermoelectric module, the resulting convective coefficients characterize the heat extraction capability of each coolant under forced-convection conditions. The calculated values are summarized in Table 4.

As shown in Table 4, the convective heat-transfer coefficient varies according to the thermophysical properties of each nanofluid, particularly thermal conductivity, viscosity, density, and specific heat. These coefficients were subsequently applied as convective boundary conditions on the upper cold-side ceramic surface of the thermoelectric generator. It is important to note that the nanofluid presenting the highest thermal conductivity or convective heat-transfer coefficient does not necessarily produce the highest thermoelectric performance. Similar behavior was reported by Cristian Francisco Ramos-Castañeda et al. [34], who found that $TiO_{2}$ provided the highest power output, efficiency, and exergy among several nanofluids despite graphene exhibiting a higher thermal conductivity. Their results indicate that thermoelectric performance depends not only on the heat extraction capability of the coolant but also on the temperature distribution established within the thermoelectric module and the resulting temperature gradients across the thermoelements. Since voltage generation is directly governed by the Seebeck effect (V = αΔT), the cooling medium that produces the most favorable temperature field within the thermoelectric elements may provide superior performance even when its convective coefficient is not the highest. Therefore, the final thermoelectric response is governed by the combined interaction between convective cooling, heat-transfer pathways, and temperature-gradient development rather than by the magnitude of the convective coefficient alone.

Although the present study employs convective boundary conditions derived from nanofluid thermophysical properties, the cooling process was represented through an equivalent convective heat-transfer coefficient applied at the cold-side ceramic surface. Therefore, the detailed fluid-flow phenomena associated with active cooling systems, including flow distribution, pump operation, temperature evolution of the coolant, and transient thermal interactions, were not explicitly modeled. Experimental investigations conducted by Selcuk Bulat et al. [41], on photovoltaic–thermoelectric systems demonstrated that the performance of thermoelectric modules depends not only on the cooling medium itself but also on the characteristics of the cooling strategy, including passive cooling, water cooling, and nanofluid-based active cooling. Their results showed that active cooling systems improve voltage, current, and power output compared with passive cooling configurations. Consequently, the present approach should be interpreted as a simplified representation of nanofluid cooling, which enables a comparative assessment of different cooling media while avoiding the additional complexity associated with a full fluid-dynamic model.

### 2.7. Boundary Conditions

The boundary conditions are defined to reproduce the steady-state operating conditions of the thermoelectric generator and to ensure consistency with well-established thermoelectric numerical frameworks. The applied thermal and electrical conditions follow the methodology commonly adopted in thermoelectric finite-element simulations and are consistent with those reported by Ramos-Castañeda et al. [34], ensuring the model gives reliable results.

From a thermal standpoint, a uniform heat flux q” is imposed at the bottom surface of the thermoelectric generator, representing the hot-side heat source. This boundary condition provides a controlled and repeatable thermal input to the system and has been widely employed in previous numerical investigations of thermoelectric generators [27]. The magnitude of the applied heat flux is defined according to the operating conditions considered in this study.

The upper surface of the thermoelectric generator, corresponding to the cold side, is maintained at a constant temperature of

$$T_{c}=25\;{}^{\circ}C$$

This represents a reference ambient condition commonly assumed in thermoelectric performance analyses [33]. This condition allows a well-defined temperature gradient to be established across the thermoelectric legs and facilitates direct comparison with results reported in the literature.

All remaining external surfaces of the thermoelectric module are assumed to be thermally insulated to minimize lateral heat losses and enforce predominantly one-dimensional heat transfer across the device thickness. Thermal contact resistance between the thermoelectric legs, metallic interconnects, and ceramic substrates is neglected to reduce model complexity and to focus on the intrinsic thermoelectric response of the system. Radiative heat-transfer effects are also ignored, as their contribution is relatively small within the operating temperature range considered. The Thomson effect is included in the simulations to capture the complete thermoelectric behavior, in accordance with previously reported formulations [34].

From an electrical standpoint, the thermoelectric module was analyzed under open-circuit conditions. To obtain a unique numerical solution of the coupled thermoelectric equations, an electrical reference potential was prescribed at one electrode of the module. Following the numerical implementation reported by Ramos-Castañeda et al. [34], a reference electric potential of:

ϕ=0.08 V

The electric potential boundary conditions were defined as V = 0 V at the n-type copper electrode and V = 0.08 V at the p-type copper electrode. These prescribed values do not correspond to an externally applied voltage source but are introduced as numerical reference conditions for the electric potential field in the finite-element formulation. Therefore, the imposed boundary conditions do not affect the potential difference generated by the thermoelectric effect.

The opposite electrode was left electrically floating, allowing the electric-potential distribution to develop naturally because of the temperature gradient imposed across the thermoelectric legs through the Seebeck effect. Therefore, the voltage reported in this work corresponds to the potential difference generated by the thermoelectric conversion process and is independent of the absolute value of the prescribed reference potential. All remaining external surfaces were considered electrically insulated to avoid parasitic current leakage and to ensure that charge transport occurred exclusively through the thermoelectric circuit.

#### Influence of Thermal and Electrical Contact Resistances in Segmented Skutterudite/$Bi_{2}Te_{3}$ Thermoelements

As described in Section 2, the thermoelectric module consists of segmented p-type and n-type thermoelements, solder layers, copper interconnectors, and alumina ceramic substrates. Consequently, several interfaces are present throughout the assembly, including skutterudite/$Bi_{2}Te_{3}$, $Bi_{2}Te_{3}$/solder, solder/copper, and copper/alumina junctions. Under practical operating conditions, these interfaces introduce thermal and electrical contact resistances that may affect heat transfer and voltage generation [42,43].

Thermal contact resistance produces temperature drops at the interfaces, reducing the effective temperature difference available across the segmented thermoelements. Since the generated voltage is directly proportional to the temperature gradient through the Seebeck effect, a reduction in the effective temperature gradient decreases the generated electromotive force (EMF) and consequently the attainable voltage output [42,44].

Similarly, electrical contact resistance increases the overall electrical resistance of the module and introduces an additional voltage loss:

$$\Delta V_{\mathrm{contact}}=IR_{\mathrm{contacts}}$$

where I is the electrical current and $R_{\mathrm{contacts}}$ is the equivalent contact resistance. Consequently,

$$V_{\mathrm{real}}<V_{\mathrm{model}}$$

because part of the generated electrical potential is dissipated at the interfaces before reaching the external terminals. Therefore, thermal contact resistances reduce the electromotive force generated inside the module, whereas electrical contact resistances reduce the useful voltage delivered to the external load. As a result, the voltage under load, supplied current, maximum electrical power, and overall thermoelectric performance may be overestimated when contact resistances are neglected [42,43,45].

In the present investigation, thermal and electrical contact resistances were intentionally neglected to isolate the influence of thermoelement geometry and segmentation on temperature distribution and voltage generation and to reduce model complexity. Consequently, the reported voltage values should be interpreted as idealized predictions corresponding to perfect interfacial conditions. Under practical operating conditions, the experimentally measured voltage is expected to be slightly lower due to additional thermal and electrical losses occurring at the skutterudite/$Bi_{2}Te_{3}$, $Bi_{2}Te_{3}$/solder, solder/copper, and copper/alumina interfaces [42,45]. Future studies may incorporate experimentally measured contact resistances to evaluate their influence under convective cooling conditions.

### 2.8. Model Validation

Figure 6, Figure 7 and Figure 8 presents the results reported by Ali and Moria [33], obtained through numerical simulations for a configuration with geometric parameters $A_{w}=0.1\;mm$, $t_{l}=0.2$ mm, and $h_{r}=0.6$. In their study, the heat flux applied to the hot side of the thermoelectric generator (TEG) ranged from 5000 to $35,000\;W/m^{2},$ while the cold-side temperature was maintained constant at 25 °C. Additionally, the effect of the number of sinusoidal cycles (N=1–4) was evaluated under steady-state conditions.

Figure 6b, Figure 7b and Figure 8b, shows the results obtained using the numerical model proposed in this work under the same operating conditions. The comparison between both figures reveals excellent agreement between the reference data and the numerical predictions, validating the proposed methodology and confirming its capability to reproduce the thermoelectric behavior of the system accurately. Furthermore, the results indicate that hollow-leg configurations exhibit superior performance and that increasing the number of sinusoidal cycles enhances both the output power and the energy conversion efficiency. In particular, the configuration with N=4 achieved the highest value across the entire range of heat fluxes analyzed.

The percentage difference between the literature data and the present results was calculated using Equation (14)

(18) $$\mathrm{Difference}\;(\%)=\frac{\left|V_{Present}-V_{Aly\&Moria}\right|}{V_{Aly\&Moria}}\times 100$$

Table 5 presents a quantitative comparison between the voltage values predicted by the present numerical model and the FEM results reported by Li and Moria. The mean relative difference between both datasets is approximately 4.33%, with most cases exhibiting deviations below 5%. The maximum discrepancies are observed at low cycle numbers and intermediate heat-flux conditions, while the differences decrease as the heat flux and number of sinusoidal cycles increase. These results demonstrate the capability of the numerical model to capture the thermal and thermoelectric response under the operating conditions investigated, supporting its application in the subsequent parametric analysis.

To evaluate the behavior of the electrical power generated by the thermoelectric device, a comparison was performed between the results obtained using the finite element method (FEM) reported by Aly and Moria and those obtained in the present work. The comparison was carried out for different values of N and heat fluxes of 5000, 15,000, 25,000, and 35,000 W/m^2^. The plots show the evolution of the thermoelectric power, $P_{TEG}$, while the table presents the corresponding numerical values, allowing the similarity between both sets of results to be directly observed.

The efficiency curves illustrate the thermoelectric performance of the analyzed configurations as a function of the operating conditions. The results enable a direct comparison of their energy conversion capabilities.

A strong agreement between the present finite-element results and the reference data confirms the accuracy of the proposed numerical model.

## 3. Results

The present analysis was performed under steady-state operating conditions; therefore, transient effects were neglected. All results were obtained using a non-uniform tetrahedral finite element mesh consisting of 146,968 elements. A mesh convergence study demonstrated that, beyond this mesh density, the variation in the generated voltage became negligible, with changes occurring only beyond the fourth decimal place. This confirms that the solution is mesh-independent and ensures an adequate resolution of the coupled thermoelectric fields within the complex hollow sinusoidal geometry of the thermoelectric generator.

The results are first presented using the hollow thermoelectric leg geometry proposed by Ali and Moria [33]. However, in the present study, a cooling strategy based on different nanofluids is implemented instead of the idealized constant cold-side temperature of 25 °C adopted in their work. This approach enables evaluation of the thermoelectric generator (TEG) performance under cooling conditions relevant to practical applications.

Figure 9 illustrates the temperature distribution throughout the entire TEG as well as within its internal region, considering the geometric parameters presented in Figure 1, the boundary conditions described in Figure 2, and the mesh discretization shown in Figure 4. For this validation case, values of dr=0.28 mm and D=0.50 mm were employed, corresponding to the configuration that enabled validation of the geometry against the results reported in the literature [33]. As shown in Figure 9, the temperature distribution exhibits a well-defined thermal gradient along the thermoelectric elements, with a smooth and continuous transition from the hot side to the cold side of the device. This behavior indicates efficient heat transfer through the segmented hollow sinusoidal legs and confirms proper thermal coupling between the thermoelectric materials. The absence of abrupt temperature discontinuities further suggests stable thermal conduction across the entire thermoelectric structure.

The temperature distribution exhibits a well-defined gradient along the thermoelectric legs, which is essential for generating Seebeck voltage. The adoption of a sinusoidal hollow geometry increases the effective heat-transfer path, enhancing the thermal resistance and promoting a more uniform temperature profile along the legs. This behavior contributes to improved thermoelectric performance, particularly in configurations with a higher number of sinusoidal cycles.

### 3.1. Physical Mechanisms of Heat Transfer and Voltage Generation

As illustrated in the proposed configuration Figure 9a,b the segmented thermoelements consist of a lower skutterudite section and an upper $Bi_{2}Te_{3}$ section connected through a hollow sinusoidal geometry. Heat is transferred from the hot ceramic substrate through the lower skutterudite segment, across the segmented interface, and subsequently through the upper $Bi_{2}Te_{3}$ segment before reaching the cold side. Unlike conventional straight thermoelements, the sinusoidal profile forces the heat flux to follow a longer and non-linear conduction pathway. Consequently, the effective conduction length becomes larger than the physical height of the thermoelement, increasing the effective thermal path available for heat transfer. Furthermore, the hollow architecture reduces the effective conduction area while increasing the conduction length. According to Fourier’s law, this combination promotes the development of larger temperature differences along the thermoelement and favors the establishment of stronger thermal gradients between the hot and cold regions [33].

The local temperature distribution is also affected by the sinusoidal geometry. Regions with smaller local cross-sectional areas constrain heat conduction and consequently produce steeper temperature gradients, whereas wider sections distribute the heat flux over a larger area and reduce local thermal gradients. This behavior generates alternating regions of high and low temperature gradients along the thermoelement length. Increasing the wave amplitude and the number of sinusoidal cycles further extends the effective thermal path and enhances the overall temperature gradient developed within the thermoelement. These observations are consistent with the findings reported by Ali and Moria [33].

From an electrical perspective, the generated voltage depends directly on the temperature gradient established along with the thermoelement through the Seebeck effect. The hollow sinusoidal geometry modifies the thermal pathway by increasing the effective conduction length while reducing the effective conduction area. Although the reduction in cross-sectional area tends to increase the electrical resistance of the thermoelement, the larger temperature difference developed along the sinusoidal pathway promotes a stronger Seebeck response. Consequently, the enhancement of the temperature gradient becomes the dominant mechanism explaining the higher voltage values obtained for larger wave amplitudes and more pronounced sinusoidal geometries. The segmented Skutterudite/$Bi_{2}Te_{3}$ configuration further contributes to this behavior. The lower skutterudite segment operates in the higher-temperature region, while the upper Bi_2_Te_3_ segment remains closer to its optimum operating range. Such a temperature distribution allows each material to contribute more effectively to thermoelectric conversion, improving the utilization of the available thermal gradient. Therefore, the combined effects of segmentation, hollow architecture, and sinusoidal geometry promote stronger Seebeck voltage generation within the proposed thermoelectric module. Overall, the voltage enhancement observed in the present study results from the combined interaction of an elongated heat-transfer pathway, modified temperature distribution, larger local temperature gradients, and more effective utilization of the thermal field through material segmentation. Consequently, the proposed hollow sinusoidal thermoelements generate higher voltages than conventional straight thermoelements operating under similar thermal conditions.

### 3.2. Performance of the Thermoelectric Generator for N Values from 1 to 4 Using Different Nanofluids Under Variable Heat Flux Conditions

This section presents the numerical analysis of the thermoelectric generator (TEG) based on the geometry obtained using the Equation (1) and the operating conditions illustrated in Figure 2. According to Ref. [33], the lower surface of the device corresponds to the hot side. It is subjected to a variable heat flux ranging from 5000 to $35,000\;W/m^{2},$ while the upper surface, corresponding to the cold side, is maintained at a constant temperature of 25 °C. Although this approach allows the thermoelectric behavior to be evaluated under idealized conditions, it does not accurately represent the actual heat dissipation mechanisms encountered in practical applications.

For this reason, the thermal load applied to the hot side of the module was kept constant throughout the study. In contrast, the fixed-temperature boundary condition imposed on the cold side was replaced by a convective cooling condition characterized by a heat transfer coefficient corresponding to each cooling fluid. The convective coefficients used in the simulations were determined from the thermophysical properties of the nanofluids described in Section 2.6 and are presented in Figure 10.

Figure 10 illustrates the temperature distribution on the upper surface of the thermoelectric module for distilled water and nanofluids based on $Al_{2}O_{3},Fe_{3}O_{4},TiO_{2}$, and graphene. The results indicate that the heat dissipation capability strongly depends on the convective heat transfer coefficient associated with each cooling fluid, leading to noticeable differences in the surface temperature attained by the module. Nanofluids exhibiting superior heat transfer characteristics promote more effective heat removal from the cold side, resulting in lower surface temperatures and potentially enhancing the temperature gradient across the thermoelectric device.

These findings are consistent with previous studies reporting improved thermal performance of nanofluids due to their enhanced effective thermal conductivity and additional energy transport mechanisms associated with suspended nanoparticles. Consequently, nanofluid-based cooling represents a promising approach for improving thermal management and enhancing the performance of thermoelectric systems operating under convective cooling conditions.

The heat transfer coefficient used in each simulation was determined from the thermophysical properties of the nanofluids described in Section 2.6 Previous studies have shown that nanofluids can enhance heat transfer due to their improved thermal conductivity and additional energy transport mechanisms, resulting in more efficient heat removal and improved thermal management.

The performance of a thermoelectric generator is directly governed by the temperature difference established between the hot and cold sides, since the voltage generated by the Seebeck effect is proportional to the thermal gradient developed across the thermoelectric legs. Consequently, any strategy capable of increasing or preserving this temperature gradient can contribute to higher electrical power generation and improved conversion efficiency.

In the case of sinusoidal configurations, increasing the geometric parameter N modifies both the thermal conduction path and the electrical transport path. The wavy geometry increases the effective leg length and the available surface area for heat transfer, promoting a more uniform temperature distribution while facilitating the establishment of larger thermal gradients. These effects become particularly significant when convective cooling with nanofluids is employed, since the enhanced heat extraction at the cold side helps maintain a greater temperature difference throughout the device.

To evaluate the thermoelectric response of the different geometrical configurations, a coupled thermal–electric steady-state model was employed, simultaneously solving the temperature and electric potential fields. The main assumptions and boundary conditions adopted in the simulations are summarized as follows:

- Perfect thermal and electrical contact were assumed between all material interfaces; therefore, interfacial thermal resistance and electrical contact resistance were neglected.
- A uniform heat flux was applied to the bottom surface of the module to represent the heat source, considering values between 5000 and $35,000\;W/m^{2}.$
- Heat dissipation from the upper surface was modeled using a convective boundary condition defined by the heat transfer coefficient corresponding to each nanofluid under investigation.
- Radiative heat losses were neglected due to their relatively small contribution compared with conductive and convective heat transfer mechanisms.
- All external surfaces were assumed to be electrically insulated, except for those designated as electrical terminals.
- An electrical reference potential was prescribed following the methodology described in Section 2.7, while the opposite electrode remained electrically floating to allow the Seebeck voltage to develop naturally.
- Seebeck, Peltier, and Thomson effects were included through temperature-dependent thermoelectric material properties implemented within the coupled-field formulation.

Although the constant cold-side temperature condition (T=25 °C) represents an idealized scenario that provides the highest output voltage in all cases, the results presented in Figure 11 show that implementing convective boundary conditions using nanofluids enables performance levels that approach this theoretical limit. As observed, the voltage corresponding to the ideal 25 °C condition remains slightly higher; however, the difference between this case and those employing advanced cooling media is minimal, particularly for larger values of N. For a heat flux of $25,000\;W/m^{2},$ the maximum output voltage under the ideal condition is approximately between 0.31 and 0.32 V for N=4, whereas nanofluids such as $TiO_{2}$ and graphene-based suspensions achieve values that are only a few millivolts lower. Figure 11 illustrates the effect of the number of revolutions (N) on the output voltage for the different cooling fluids considered. An increase in output voltage is observed as N increases for all cooling fluids and heat flux conditions. However, the voltage gain gradually diminishes at higher values of N. Although N = 4 yielded the highest output voltage, the improvement relative to N = 3 was marginal. This behavior suggests diminishing performance returns, were further increases in geometric complexity result in only limited enhancements in thermoelectric performance. Consequently, N = 3 was identified as the most suitable configuration, providing nearly the same performance as N = 4 while maintaining a simpler geometry and lower computational cost. Therefore, N = 3 was selected as the reference configuration for all subsequent analyses.

A similar trend is observed at higher heat fluxes, where the ideal condition yields the highest voltage levels, and the best-performing nanofluids closely follow without surpassing it. These small differences demonstrate that enhanced convective cooling significantly reduces the performance gap with respect to the idealized boundary condition.

It should be noted that all results discussed in this section were obtained using a reference sinusoidal geometry characterized by a revolution distance of $d_{r}=0.28$ mm and a separation between thermoelectric elements of D=0.50 mm. These dimensions were selected as the baseline design for assessing the impact of different cooling strategies. Moreover, this reference configuration serves as a starting point for future geometric optimization studies aimed at evaluating the effects of variations in the revolution distance and the spacing between adjacent thermoelectric elements on the temperature distribution, voltage generation, power output, and overall thermoelectric performance of the device.

### 3.3. Effect of Revolution Distance ($d_{r}$) on the Output Voltage of the Thermoelectric Generator for the N = 3 Sinusoidal Configuration

As discussed in Section 3.2, two important geometric parameters of the TEG were identified: the revolution distance $\left(d_{r}\right)$ and the spacing between thermoelements (D). Although the parameter dr was not reported by Aly and Moria [33], the values dr=0.28 mm and D=0.5 mm were adopted for the validation performed in this work.

Based on these considerations, this section evaluates the effect of the distance of revolution ($d_{r}$), taking 0.15 mm as the lower limit. This value corresponds to the minimum allowable distance between the axis of revolution and the profile defined by Equation (1). As illustrated in Figure 10, values of $d_{r}$ below 0.15 mm cause the axis of revolution to intersect the generated profile, preventing proper construction of the three-dimensional geometry. Therefore, $d_{r}$ =0.15 mm was adopted as the minimum feasible value for parametric analysis.

Figure 12 shows that the output voltage decreases continuously as the revolution distance, dr, increases from 0.15 to 0.58 mm. The increase in dr enlarges the internal cavity of the sinusoidal thermoelectric leg, modifying the heat-transfer path and reducing the effective thermoelectric material participating in energy conversion. As a result, the voltage generated by the Seebeck effect decreases for all analyzed cooling conditions. The ${TiO}_{2}$ nanofluid consistently provides the highest voltage output, whereas the water-cooling case exhibits the lowest values. Despite these differences, the nearly parallel behavior of the curves indicates that the geometric parameter $d_{r}$ governs the overall performance trend of the device. Since the thermoelement spacing was maintained constant at D=0.5 mm throughout the study, the observed reduction in voltage can be directly attributed to the increase in revolution distance $d_{r}$.

Changing $d_{r}$ modifies the geometry of the thermoelements and, consequently, the volume and internal electrical resistance of the TEG. The corresponding values for the geometries compared in this study are reported in Table 6.

Figure 13 shows that the revolution distance $d_{r}$ has a significant nonlinear influence on the electrical performance of the sinusoidal thermoelectric generator. For all cooling conditions, increasing $d_{r}$ reduces the output voltage, output power, and conversion efficiency. Under Ti$O_{2}$ nanofluid cooling, for example, increasing $d_{r}$ from approximately 0.15 to 0.58 mm decreases the output voltage from approximately 0.367 to 0.225 V and the electrical power from approximately 0.109 to 0.050 W, corresponding to reductions of about 39% and 54%, respectively. Over the same range, the conversion efficiency decreases from approximately 26.5% to 12.4%. Similar trends are obtained for water and air cooling, although at lower absolute performance levels.

The cooling condition also substantially affects the thermoelectric response. At $d_{r}\approx 0.15$ mm, Ti$O_{2}$ nanofluid cooling produces approximately 0.367 V and 0.109 W, compared with approximately 0.295 V and 0.080 W for water and 0.275 V and 0.046 W for air. Therefore, the Ti$O_{2}$ nanofluid increases the output power by approximately 36% relative to water and by more than 100% relative to air in this geometrical condition. The corresponding efficiencies are approximately 26.5%, 23%, and 10.4% for Ti$O_{2}$, water, and air, respectively. These results demonstrate that thermoelectric performance is governed by the combined effects of geometrical optimization and heat rejection at the cold side. A smaller $d_{r}$ favors the investigated sinusoidal geometry, while enhanced cooling further increases the effective temperature difference across the thermoelectric elements. Notably, the response is nonlinear, with the strongest sensitivity occurring at small $d_{r}$, indicating that careful geometrical control is particularly important within this region.

### 3.4. Effect of Convective Cooling Conditions on Thermoelectric Output Voltage

Based on the results obtained in Section 3.2, the optimal geometric configuration corresponded to a revolution distance of ${TiO}_{2}$ $d_{r}\;=0.15\;mm$ and a thermoelement spacing of D=0.5 mm, which yielded the highest output voltage. Using this configuration, the influence of the applied heat flux was analyzed for values ranging from 5000 to $35,000\;W/m^{2}$. The performance of the thermoelectric generator was evaluated under three cold-side cooling strategies: forced air, water, and a ${TiO}_{2}$-based nanofluid.

The analysis reveals that, for all cooling conditions, the output voltage increases monotonically with increasing heat flux as a direct consequence of the enhanced temperature gradient across the thermoelectric legs. Among the evaluated cooling media, the ${TiO}_{2}$ nanofluid consistently yields the highest voltage values, followed by water and air. As illustrated in Figure 14, the output voltage increases monotonically with increasing heat flux for all cooling conditions due to the corresponding enhancement of the temperature gradient across the thermoelectric legs. Among the cooling strategies evaluated, the ${TiO}_{2}$ nanofluid consistently yields the highest voltage values, followed by water and air. This behavior is attributed to the superior thermal properties of the nanofluid, which enhance heat removal at the cold side and promote a larger effective temperature difference across the thermoelectric elements. Despite the differences in absolute voltage levels, the similar slopes observed for all cooling conditions indicate that the cooling mechanism primarily acts as a scaling factor, without significantly altering the overall response to the applied heat flux. These results confirm that, for the geometry under investigation, the applied heat flux is the dominant parameter governing voltage generation, while enhanced convective cooling, particularly with ${TiO}_{2}$ nanofluids, improves thermoelectric performance under operating conditions.

To further interpret these results, it is important to consider the thermal–electrical coupling governing the system. As the heat flux increases, the amount of thermal energy available at the hot side rises, leading to a larger temperature difference across the thermoelectric legs and, consequently, to a higher Seebeck voltage. However, the effectiveness of this process strongly depends on the ability of the cooling mechanism to dissipate heat on the cold side. In this context, the improved performance observed with ${TiO}_{2}$ nanofluid cooling can be associated with enhanced convective heat transfer, which maintains a lower cold-side temperature even under high thermal loads. This effect becomes more pronounced at higher heat flux values, where the limitations of conventional cooling media such as air and water become evident. As shown in the temperature distributions, the ${TiO}_{2}$ case exhibits a more uniform and lower temperature profile, indicating more efficient heat extraction. From a practical standpoint, these results suggest that while increasing heat flux is an effective strategy to boost thermoelectric output, its benefits can only be fully exploited when coupled with advanced cooling solutions. Therefore, integrating nanofluid-based cooling systems is essential for maintaining high performance and thermal stability in thermoelectric devices operating under high heat flux conditions.

### 3.5. Electric Field Distribution as a Function of Revolution Distance

To evaluate the effect of the revolution distance on the electrical behavior of the thermoelectric elements, the spatial distribution of the electric field intensity was analyzed for different geometric configurations. The electric field distribution is directly related to the spatial variation of electric potential generated by the Seebeck effect and provides qualitative information regarding the electrical response of the thermoelements. Therefore, the analysis is useful for identifying regions of electric-field concentration and for understanding how geometric modifications influence the electrothermal behavior of the proposed configuration. However, local maxima of electric field intensity should not be interpreted as a direct measure of thermoelectric performance, since they may arise from geometrical field concentration effects and do not necessarily correspond to improvements in voltage generation or overall device efficiency.

This section presents the results obtained for two revolution distance values, $d_{r}=0.15$ mm and $d_{r}=0.28$ mm, considering in each case the extreme spacing configurations (D=0.05 mm and D=0.75 mm). The analysis focuses on the magnitude and spatial distribution of the electric field intensity throughout the thermoelectric elements and on how these distributions are affected by variations in $d_{r}$ and D.

Figure 15a shows the distribution of electric field intensity within the thermoelectric elements for a revolution distance of $d_{r}=0.15$ mm. For the configuration with D=0.05 mm, a maximum electric field intensity of 65.595 V/m was obtained, whereas increasing the revolution distance to $d_{r}=0.28$ mm resulted in a maximum field intensity of 108.53 V/m. This increase corresponds to approximately 65.5% relative to the initial configuration. Furthermore, according to the color scales presented in Figure 13a,b, the electric distribution extends over a wider region of the thermoelements as the revolution distance increases.

In both configurations, the highest electric field intensities are primarily located near the necked or constricted regions of the geometry, where abrupt changes in cross-sectional area occur. As the revolution distance increases, these regions become more pronounced and the spatial distribution of the electric field expands, particularly in the upper portions of the thermoelements. This behavior indicates that the revolution distance modifies the distribution of electric potential within the thermoelements and influences the local electric-field gradients established throughout geometry. However, these local electric-field maxima should be interpreted because of field concentration associated with the selected geometry rather than as a direct measure of thermoelectric performance. Therefore, the electric field contours are used here to illustrate changes in the electrical response of the thermoelements, while the thermoelectric performance is evaluated through the generated voltage and its relationship with the Seebeck effect.

#### Analysis of the Electric Field for $d_{r}$ = 0.28 mm

To analyze the influence of the revolution distance on the electrical response of the system, a second configuration with $d_{r}=0.28$ mm was evaluated while maintaining the same extreme spacing conditions (D=0.05 mm and D=0.75 mm). The corresponding results are presented in Figure 15a,b.

Figure 16 shows the electric field distribution within the thermoelements. For D=0.05 mm, a maximum electric field intensity of 53.651 V/m was obtained, whereas for D=0.75 mm the maximum value increased to 80.201 V/m. In both cases, the highest electric field intensities are concentrated near the constricted regions of the sinusoidal geometry, where abrupt variations in the cross-sectional area occur.

Increasing the spacing between thermoelements modifies the spatial distribution of the electric potential and consequently the electric field established within the thermoelements. However, these local electric-field maxima should be interpreted as a consequence of field concentration associated with the selected geometry rather than as a direct measure of thermoelectric performance. Therefore, the electric field contours are used to illustrate the electrical response of the proposed geometry, while the thermoelectric performance is evaluated through the generated voltage and its relationship with the Seebeck effect.

For the configuration with D=0.05 mm, a maximum electric field intensity of 53.651 V/m was obtained, whereas for D=0.75 mm, the maximum value reached 80.201 V/m. This represents an increase of approximately 49.5%. Furthermore, according to the scales shown in the simulation, the electric-field intensity range extends from $5.6258\times 10^{-11}$ V/m to 53.651 V/m for D=0.05 mm, whereas for D=0.75 mm the range increases from $5.7733\times 10^{-12}$ V/m to 80.201 V/m.

As in the previous case, the highest electric-field intensities are concentrated near the constricted regions of the sinusoidal geometry, where abrupt variations in cross-sectional areas occur. For D=0.75 mm, the maximum electric-field intensity increased from 53.651 V/m to 80.201 V/m compared with the D=0.05 mm configuration.

These results indicate that both the revolution distance and the spacing between thermoelements influence the spatial distribution of the electric field within the thermoelements. While increasing D modifies the intensity and location of local electric-field concentrations, increasing $d_{r}$ alters the spatial distribution of the electric potential throughout the geometry. However, these local electric-field maxima should be interpreted because of field concentration associated with the selected geometry rather than as a direct measure of thermoelectric performance. Therefore, the electric-field contours are used here to visualize changes in the electrical response of the thermoelements, whereas thermoelectric performance is evaluated through the generated voltage and its relationship with the Seebeck effect.

### 3.6. Combined Effect of Revolution Distance and Heat Flux on Thermoelectric Voltage Generation Under TiO_2_-Based Convective Cooling

The results demonstrate that the output voltage is strongly dependent on both the applied heat flux and the revolution distance. As the heat flux increases from 5000 to 35,000 W/m^2^, the generated voltage increases considerably due to the larger temperature gradient established across the thermoelectric legs. Conversely, increasing the revolution distance from 0.15 mm to 0.58 mm produces a continuous reduction in voltage generation. This behavior is attributed to the enlargement of the internal cavity, which reduces the effective volume of the thermoelectric material and modifies the thermal and electrical transport paths within the leg. The highest voltage value, approximately 0.39 V, is obtained for the combination of the maximum heat flux and the minimum revolution distance ($d_{r}$ = 0.15 mm), whereas the lowest voltage values occur at low heat fluxes combined with larger revolution distances. These results confirm that minimizing the revolution distance enhances thermoelectric performance and that heat flux remains the dominant driving parameter for voltage generation under ${TiO}_{2}$ nanofluid cooling conditions [37].

The contour plot in Figure 17b illustrates the combined influence of heat flux ($q^{\prime \prime }$) and revolution distance ($d_{r}$) on the output voltage (ΔV). The results indicate that the output voltage increases significantly with increasing heat flux, while it decreases as $d_{r}$ increases. The maximum voltage, approximately 0.3916 V, is achieved at a heat flux close to $35,000\;W/m^{2}$ and a revolution distance of 0.15 mm. As $d_{r}$ increases to intermediate values (0.20–0.25 mm), the voltage decreases to approximately 0.3513 V, whereas a heat flux of about $22,500\;W/m^{2}$ yields an output voltage of 0.3109 V. In the central region of the parameter space, corresponding to $q^{\prime \prime }\approx 30,000\;{W/m}^{2}$ and $d_{r}\approx 0.40\;\mathrm{mm}$, the voltage decreases to 0.2705 V. Under lower heat flux conditions, around $12,500–15,000\;W/m^{2},$ the output voltage further drops to 0.2301 V and 0.1898 V, respectively, while the minimum observed value is approximately 0.1494 V at $q^{\prime \prime }\approx 10,000\;{W/m}^{2}$ and $d_{r}\approx 0.47\;\mathrm{mm}$. These findings demonstrate that heat flux is the dominant parameter governing voltage generation, whereas larger revolution distances tend to reduce the device’s electrical performance.

### 3.7. Influence of Inter-Leg Separation Distance (D) on Device Performance with **TiO_2_** Nanofluid Convective Cooling

The revolution distance ($d_{r}$) between thermocouples was kept constant at 0.15 mm since, according to the results presented in Section 3.2, this configuration yields the maximum output voltage of the thermoelectric generator (TEG). Under this condition, the inter-leg spacing (D) was varied from 0.05 mm to 0.85 mm to evaluate its influence on the thermoelectric performance of the device. Heat dissipation was achieved through convective cooling using a ${TiO}_{2}$ nanofluid characterized by its corresponding convective heat transfer coefficient. The resulting voltage response surface is shown in Figure 18a, where the combined effects of heat flux and inter-leg spacing can be observed. This parametric analysis allowed the assessment of the effect of inter-leg spacing on both the thermal distribution and electrical response of the TEG, enabling the identification of the most favorable geometric configurations for maximizing voltage generation.

Figure 18b presents the contour map of the output voltage (ΔV) for a fixed revolution distance of $d_{r}$ = 0.15 mm under convective cooling with a ${TiO}_{2}$ nanofluid. The analysis covers heat flux values ranging from 5000 to $35,000\;W/m^{2}$ and inter-leg spacings (D) between 0.05 and 0.85 mm. Within this operating domain, the predicted output voltage varies from approximately 0.049 V to 0.38 V, with the highest values occurring at elevated heat fluxes and specific inter-leg spacings. The contour representation provides a clear visualization of the combined influence of thermal loading and geometric spacing on the electrical performance of the thermoelectric generator.

#### Analysis of Output Voltage with Respect to Inter-Leg Separation Distance and Heat Flux

Figure 19a illustrates the response surface of the output voltage (ΔV) obtained for a fixed revolution distance of $d_{r}$ = 0.28 mm, a value selected according to the reference configuration reported in the literature [33]. Under ${TiO}_{2}$-based convective cooling, the inter-leg spacing (D) was varied from 0.05 mm to 0.85 mm, while the applied heat flux ranged from 5000 to $35,000\;W/m^{2}$. The resulting surface exhibits distinct regions of voltage increase and decrease, forming pronounced valleys and peaks that reveal the combined influence of both parameters on device performance. Output voltage values range from approximately 0.029 V in the low-performance regions to 0.358 V under favorable operating conditions. In general, higher heat fluxes promote increased voltage generation. However, local drops are observed for specific values of D, indicating that the spacing between thermoelectric elements significantly affects the thermal distribution and, consequently, the electrical response of the TEG.

Figure 19a illustrates that an increase in output voltage is observed as both the heat flux and inter-leg spacing increase. For the configuration with $d_{r}$ = 0.28 mm, the maximum voltage reached approximately 0.358 V at a heat flux of 35,000 W/m^2^ and an inter-leg spacing close to 1.0 mm. However, the response surface does not exhibit purely linear responses as indicated by the inflection regions highlighted in Figure 17b, a change in the rate of voltage increase occurs for intermediate spacing values, particularly within the range of D ≈ 0.3−0.5 mm and heat fluxes above 20,000 W/m^2^. In this region, the surface curvature changes noticeably, indicating a transition from a regime of rapid voltage enhancement to one where further increases in spacing produce progressively smaller voltage gains.

The observed behavior can be attributed to the influence of geometric parameters on the distribution of electric field within the thermoelements. Increasing the inter-thermoelement spacing initially promotes a stronger electric field, enhancing voltage generation. However, the magnitude of the voltage increase indicating sensitivity of the thermoelectric response to further geometric modifications. Consequently, larger spacing values provide diminishing performance gains compared with those observed at smaller spacing intervals. See singularity points in Figure 18 and Figure 19.

## 4. Discussion

For low spacing values, small increases in D generate substantial improvements in voltage generation, whereas beyond the inflection region, the slope of the surface decreases considerably. Consequently, although the highest voltage values are obtained near D = 0.85 mm, the incremental benefit relative to intermediate spacing values become increasingly limited. The presence of these singularity regions indicates the existence of an optimal geometric range in which heat transport, charge transport, and potential electric gradients maintain a favorable balance.

The observed behavior can be attributed to the influence of geometric parameters on the distribution of electric potential within the thermoelements. Initially, increasing the inter-thermoelement spacing promotes a stronger electric potential gradient, thereby enhancing voltage generation. However, beyond a certain spacing value, the electrical response becomes progressively less sensitive to additional geometric changes, leading to a gradual saturation trend. Consequently, further increases in spacing provide only marginal improvements compared with those observed at smaller spacing intervals.

## 5. Conclusions

This work presented a comprehensive numerical investigation of a segmented thermoelectric generator employing hollow sinusoidal legs,${Bi}_{2}{Te}_{3}$–skutterudite segmented materials, and convective nanofluid-based cooling conditions. A finite-element electrothermal model was developed and successfully validated against previously reported results, showing excellent agreement with the literature. Unlike previous studies that independently evaluated thermoelectric materials, cooling strategies, or geometric optimization, the present work analyzed the coupled effects of geometric and thermal parameters on the electrothermal behavior of a non-conventional thermoelectric configuration, enabling the establishment of design criteria for maximizing electrical performance.

The results demonstrated that geometric design and cooling strategies cannot be optimized independently, since both factors are strongly coupled and jointly determine device performance. The number of sinusoidal cycles significantly influenced the thermoelectric response because the wavy geometry increased the effective thermal and electrical transport paths. Although the highest voltages were obtained for configurations with a larger number of cycles, the incremental gains became progressively smaller while geometric complexity increased. Consequently, the N=3 configuration was identified as an attractive compromise between performance enhancement and geometric simplicity.

Among the evaluated cooling conditions, the idealized constant-temperature boundary condition of 25 °C generated the highest voltages, representing the theoretical upper performance limit of the device. However, this assumption overestimates the actual thermoelectric performance achievable under operating conditions. The study revealed a strong interaction between thermal management and geometric configuration. Among the cooling alternatives, ${TiO}_{2}$-based nanofluid cooling exhibited the best performance, maintaining larger temperature gradients along the thermoelements and producing voltage values only slightly below those obtained under ideal cooling conditions. Under high thermal loads approaching 35,000 W/$m^{2}$, output voltages of up to 0.39 V were achieved, confirming the effectiveness of ${TiO}_{2}$-based cooling for preserving thermoelectric performance.

The revolution distance ($d_{r}$) was identified as one of the most influential geometric parameters. The optimum configuration was obtained at $d_{r}=0.15$ mm, whereas increasing this parameter to 0.58 mm produced a continuous reduction in voltage generation. This behavior is attributed to the enlargement of the internal cavity, which decreases the active thermoelectric material volume and weakens the thermal and electrical transport mechanisms responsible for voltage generation. The comparative analysis between the optimized geometry and the validation geometry ($d_{r}=0.28$ mm) confirmed that minimizing the revolution distance improves response of the device.

The response surface analysis further demonstrated the strong coupling between geometric and thermal parameters. Both heat flux and inter-leg spacing (D) significantly influenced voltage generation, allowing the identification of well-defined operating regions where the output voltage ranged from approximately 0.03 V to 0.39 V. The results also revealed a non-linear dependence between voltage generation and inter-leg spacing, indicating that optimal geometric configurations exist and cannot be predicted through simple linear trends.

Electric-field analysis provided additional evidence of the influence of geometric parameters on device performance. For $d_{r}=0.15$ mm, increasing the inter-leg spacing from D=0.05 mm to D=0.75 mm increases the maximum electric-field intensity from 65.60 V/m to 108.53 V/m, corresponding to a 65.5% enhancement. Similarly, for $d_{r}=0.28$ mm, the electric field increased from 53.65 V/m to 80.20 V/m, representing a 49.5% improvement. Conversely, increasing the revolution distance from 0.15 mm to 0.28 mm reduced the peak electric field by approximately 26%, demonstrating that excessive cavity expansion limits the electric potential gradients responsible for voltage generation.

Overall, the present study establishes quantitative electrothermal design criteria for hollow sinusoidal segmented thermoelectric generators operating under convective cooling conditions. The results confirm that the combination of a minimum revolution distance ($d_{r}=0.15$ mm), an appropriate inter-leg spacing, and ${TiO}_{2}$-based nanofluid cooling provides the most favorable conditions for maximizing voltage generation. These findings contribute to the design of next-generation thermoelectric generators for waste-heat recovery applications and provide practical design guidelines for enhancing thermoelectric performance under convective operating conditions. Furthermore, the methodology developed in this work establishes a robust framework for future multiparametric optimization studies involving advanced geometric configurations, innovative cooling strategies, and high-performance thermoelectric materials.

## Figures and Tables

**Figure 1:**
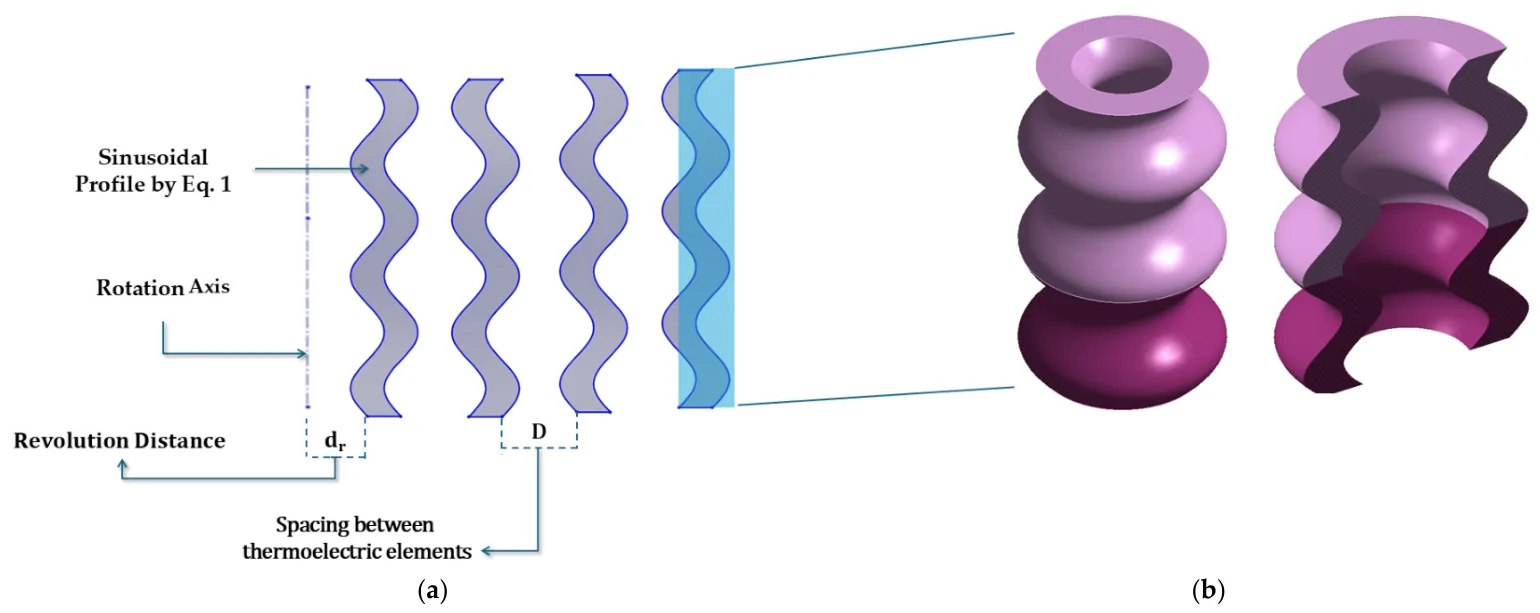
Geometry generation procedure: (**a**) sinusoidal profile and design parameters D and $d_{r}$; (**b**) hollow segmented thermoelement obtained through the revolution of the offset profile.

**Figure 2:**
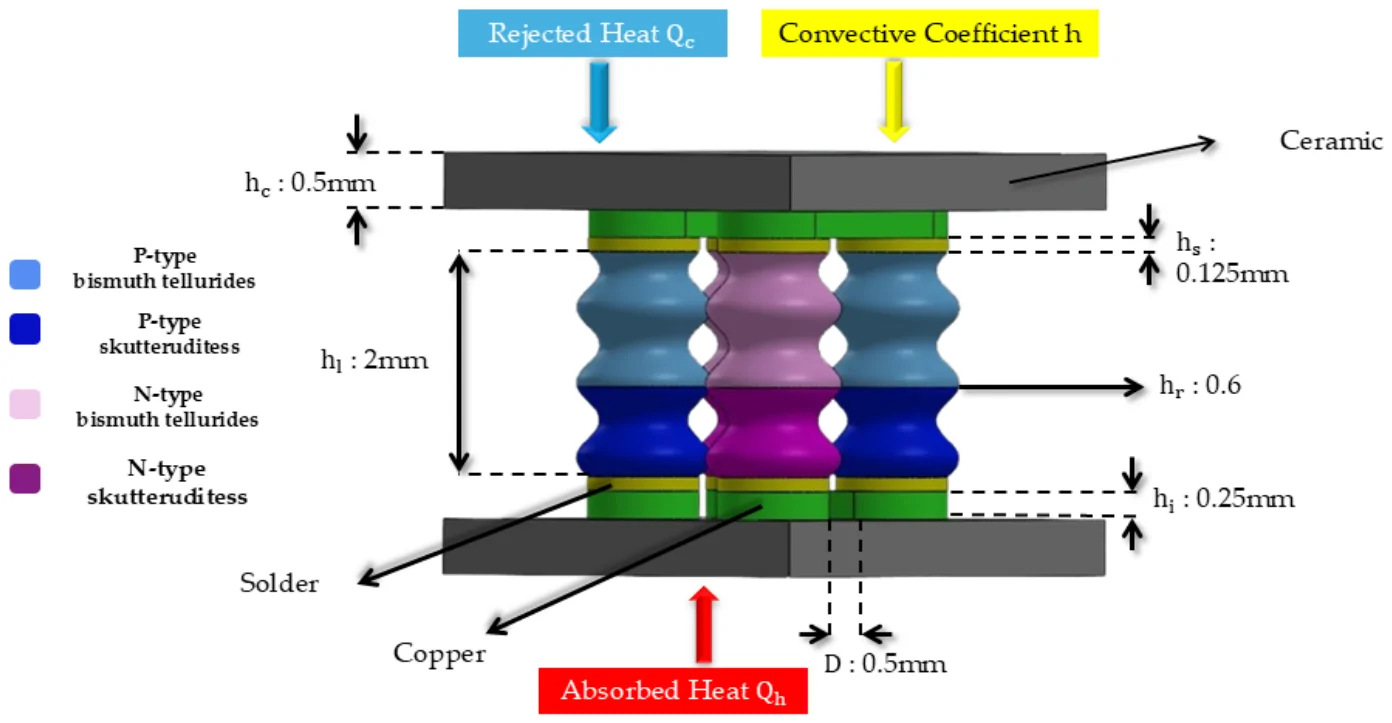
Thermoelectric generator and its geometric dimensions to N=3.

**Figure 3:**
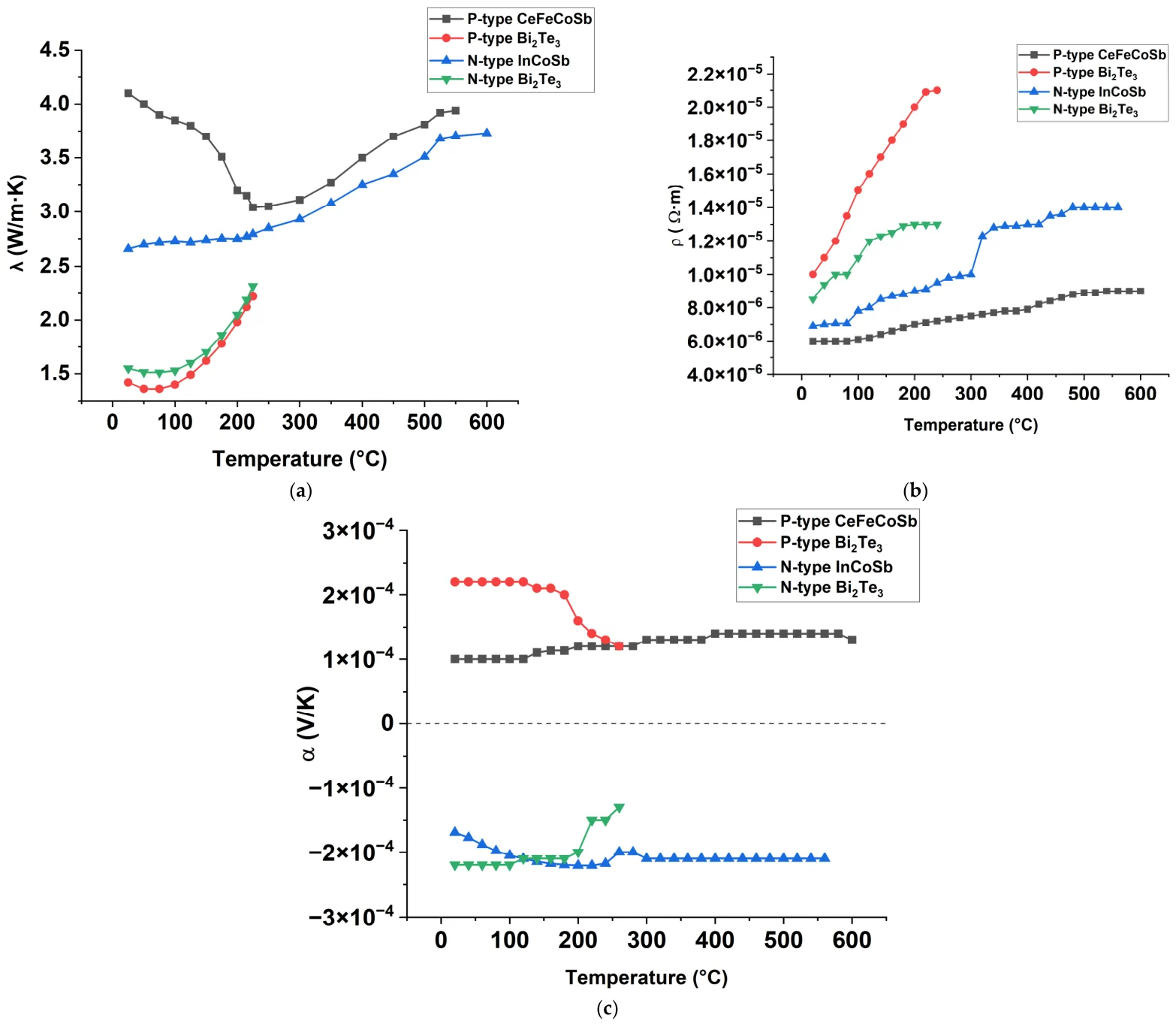
Temperature-dependent material properties: (**a**) Thermal Conductivity, (**b**) Electrical Resistivity, (**c**) Seebeck Coefficient [14,19].

**Figure 4:**
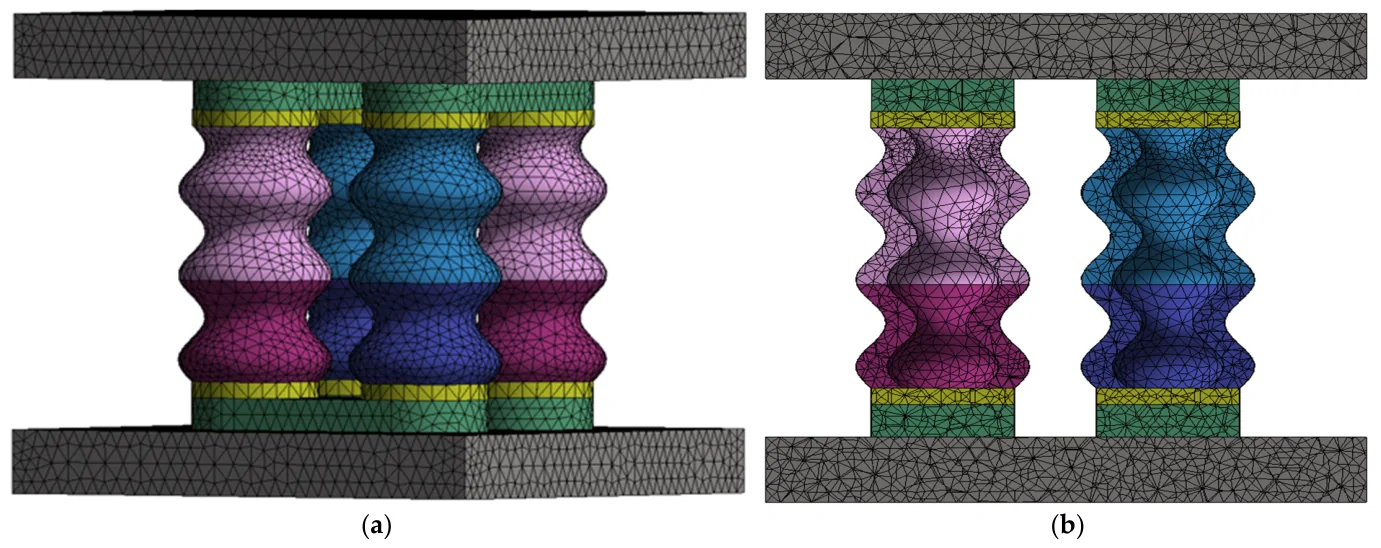
Unstructured tetrahedral finite element mesh of the segmented TEG: (**a**) full mesh of the system; (**b**) transverse cross-sectional view of the mesh [7,26].

**Figure 5:**
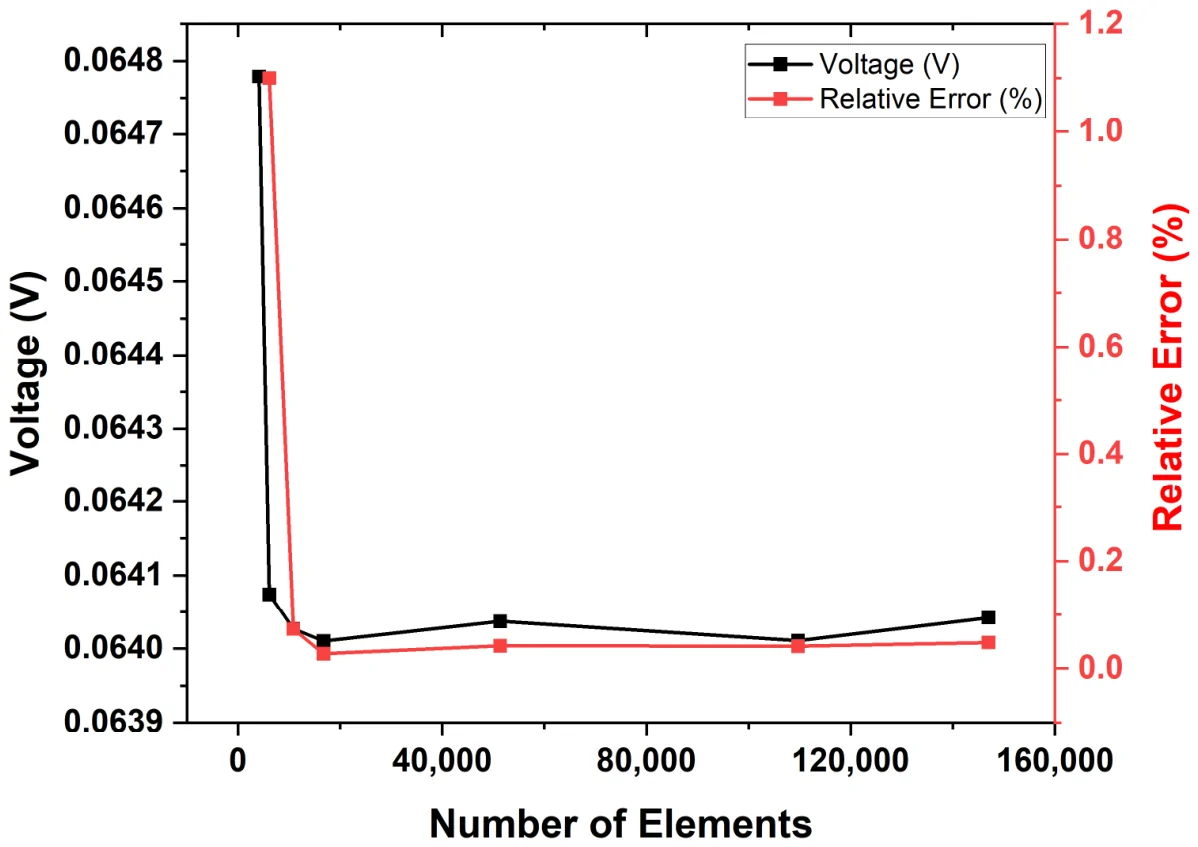
Mesh convergence study showing the variation in the predicted output voltage as a function of the total number of mesh elements.

**Figure 6:**
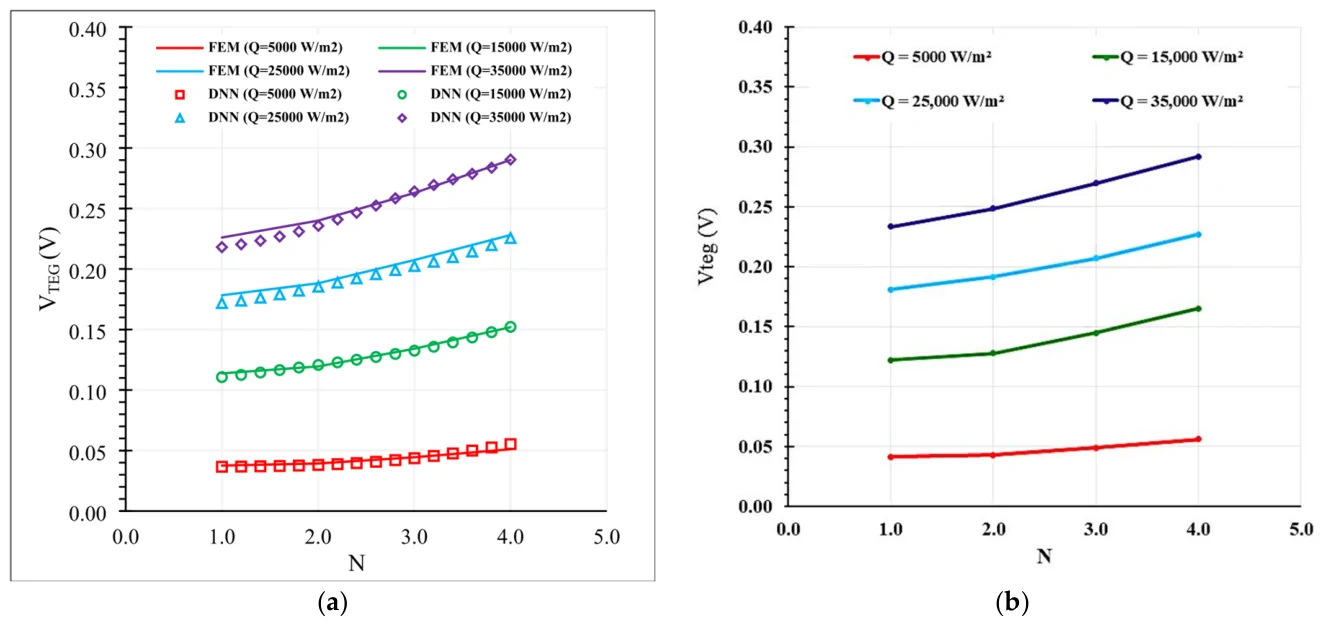
Comparison of thermoelectric performance as a function of N: (**a**) literature data from Ali and Moria [33], and (**b**) results obtained in the present study for validation.

**Figure 7:**
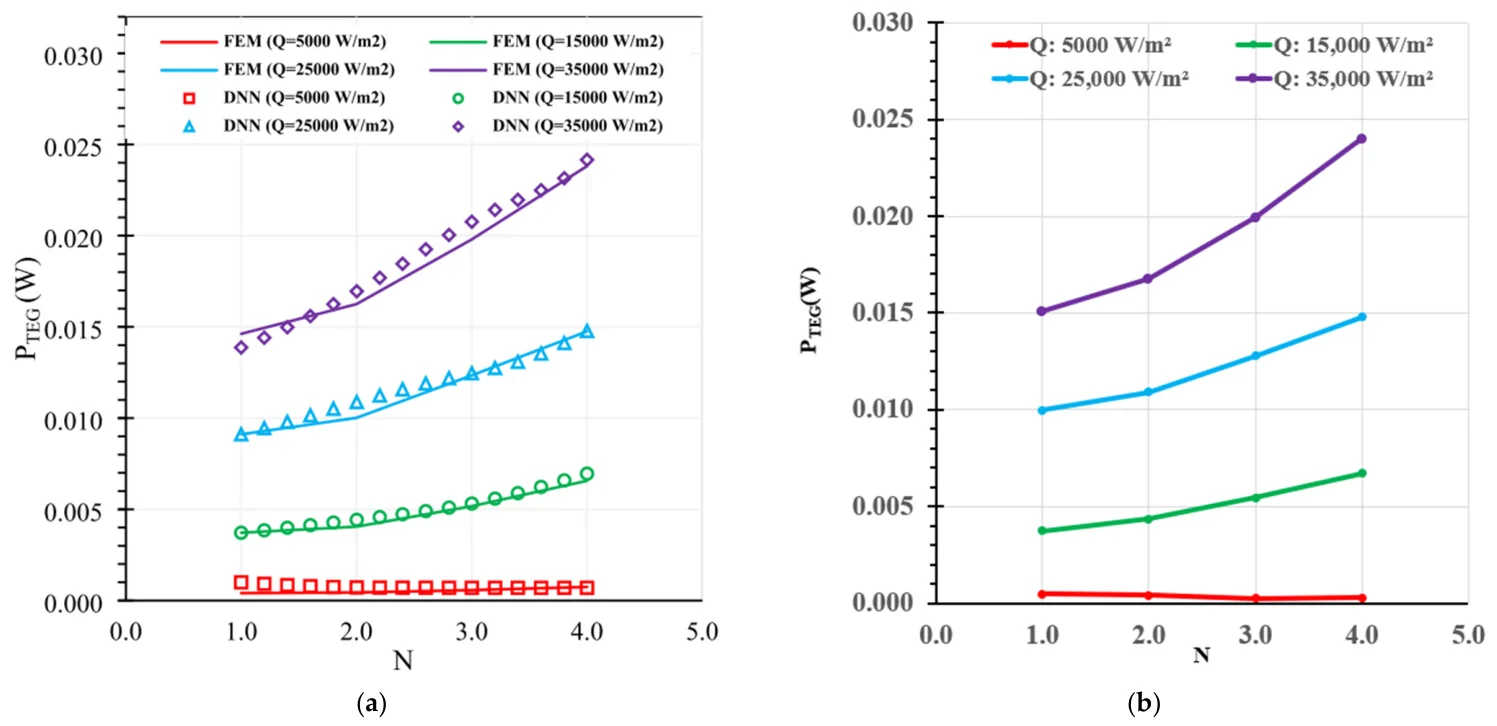
Thermoelectric power output ($P_{TEG}$) as a function of number of sinusoidal cycles (N) for different heat fluxes. (**a**) Comparison between FEM results and DNN predictions. [33] (**b**) Variation of $P_{TEG}$ with N at Q=5000, 15,000, 25,000, and $35,000\;W/m^{2}$.

**Figure 8:**
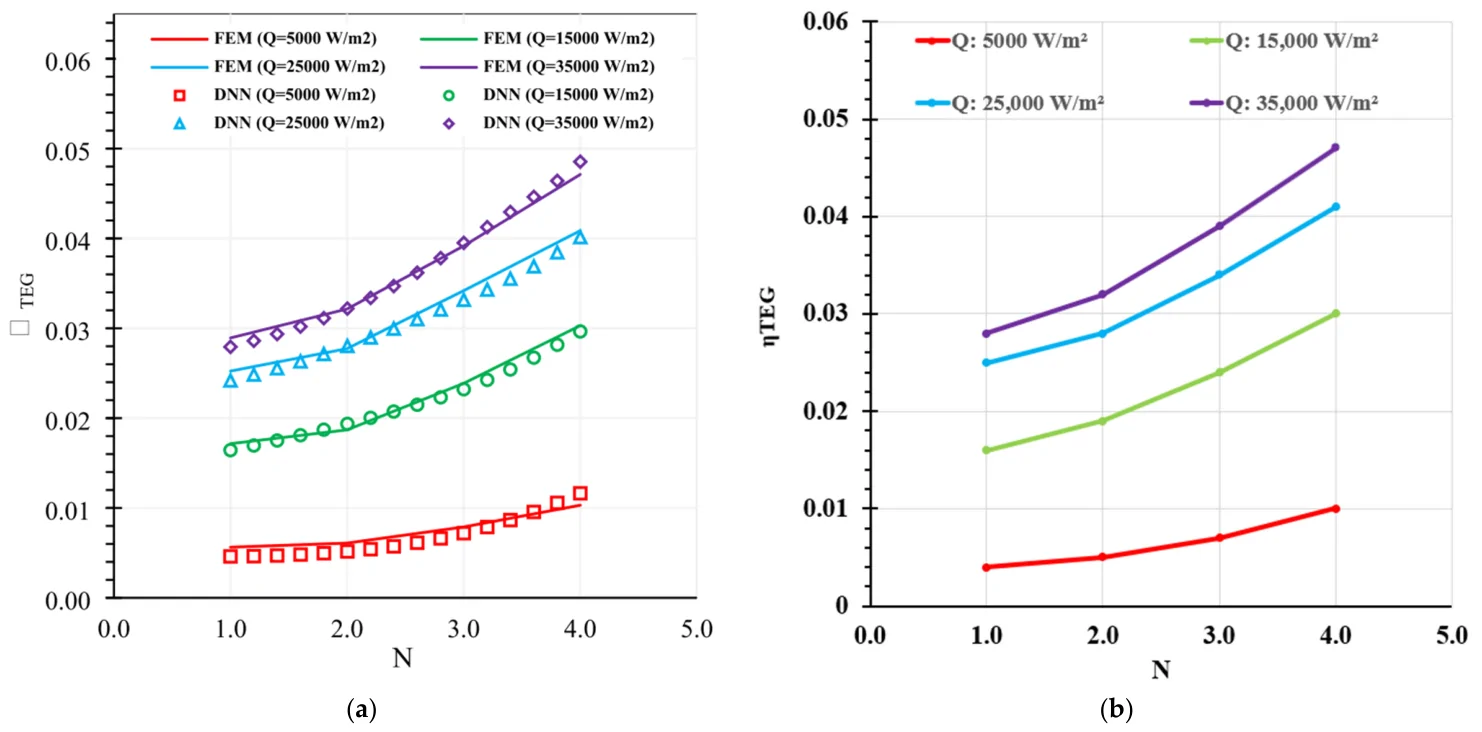
Thermoelectric efficiency (ηTEG) as a function of N for different heat fluxes: (**a**) comparison between the finite element method (FEM) and deep neural network (DNN) results [33]; (**b**) evolution of the thermoelectric efficiency obtained by FEM for Q = 5000, 15,000, 25,000, and 35,000 W/m^2^.

**Figure 9:**
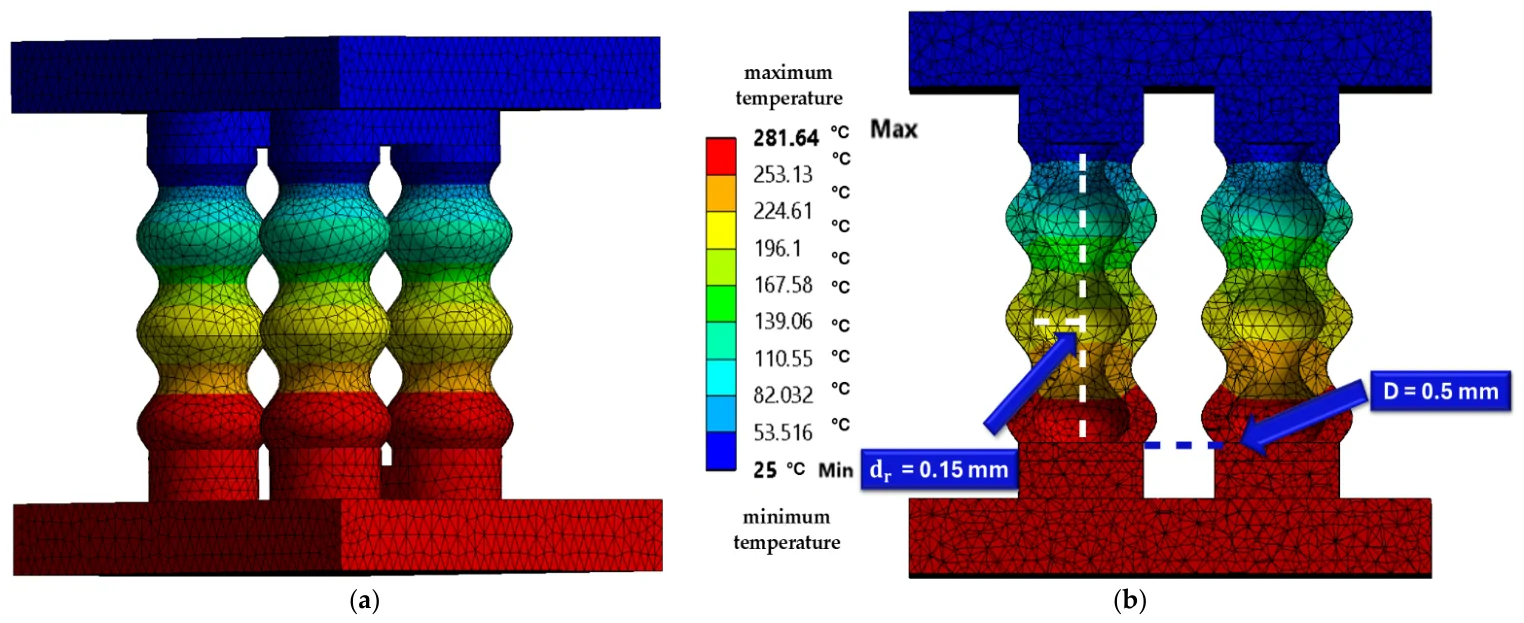
Temperature distribution in a hollow sinusoidal thermoelectric generator with N = 3: (**a**) temperature contour distribution in the complete geometry and (**b**) cross-sectional view showing the geometric parameters $d_{r}$ and D.

**Figure 10:**
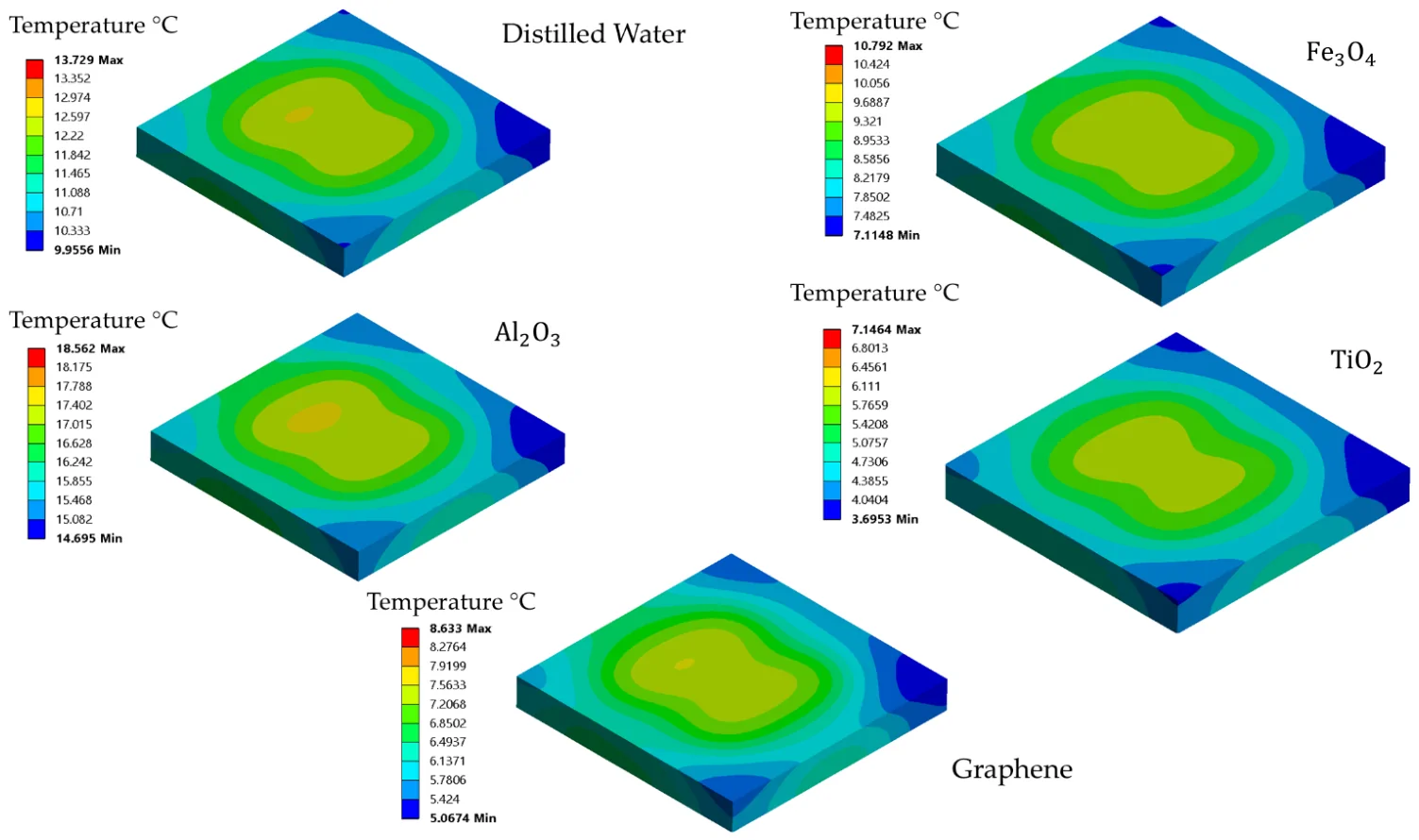
Effect of Convective Cooling Fluids on Surface Temperature Distribution.

**Figure 11:**
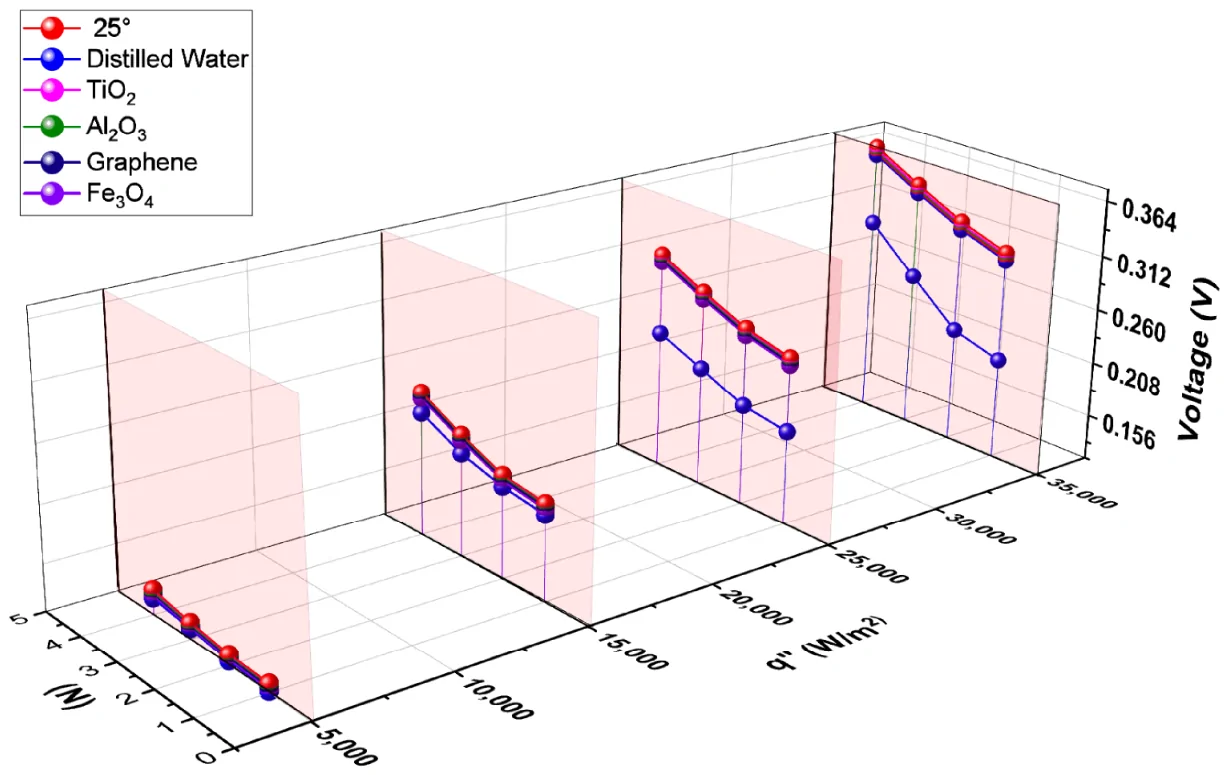
Simulated output voltage of the system as a function of the number of thermoelectric elements (N=1, 2, 3, 4) under different heat flux $\left(q^{\prime \prime }\right)$ conditions; $d_{r}$ = 0.15 mm and D = 0.5 mm.

**Figure 12:**
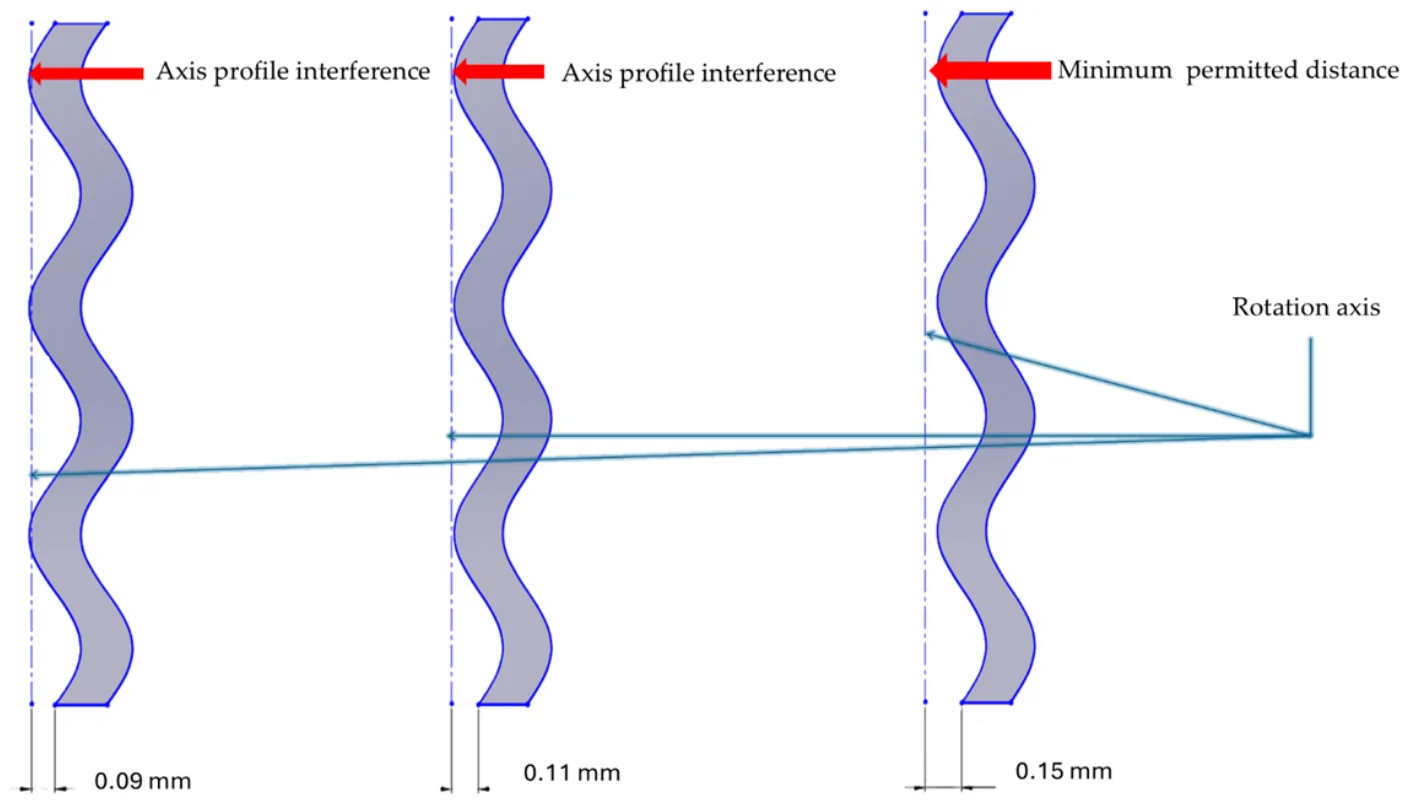
Effect of the revolution distance $d_{r}$ on the geometric feasibility of the sinusoidal thermoelectric leg. The minimum allowable distance is $d_{r}=0.15$ mm. For $d_{r}<0.15$ mm, the axis of revolution intersects the sinusoidal profile, resulting in an invalid geometry.

**Figure 13:**
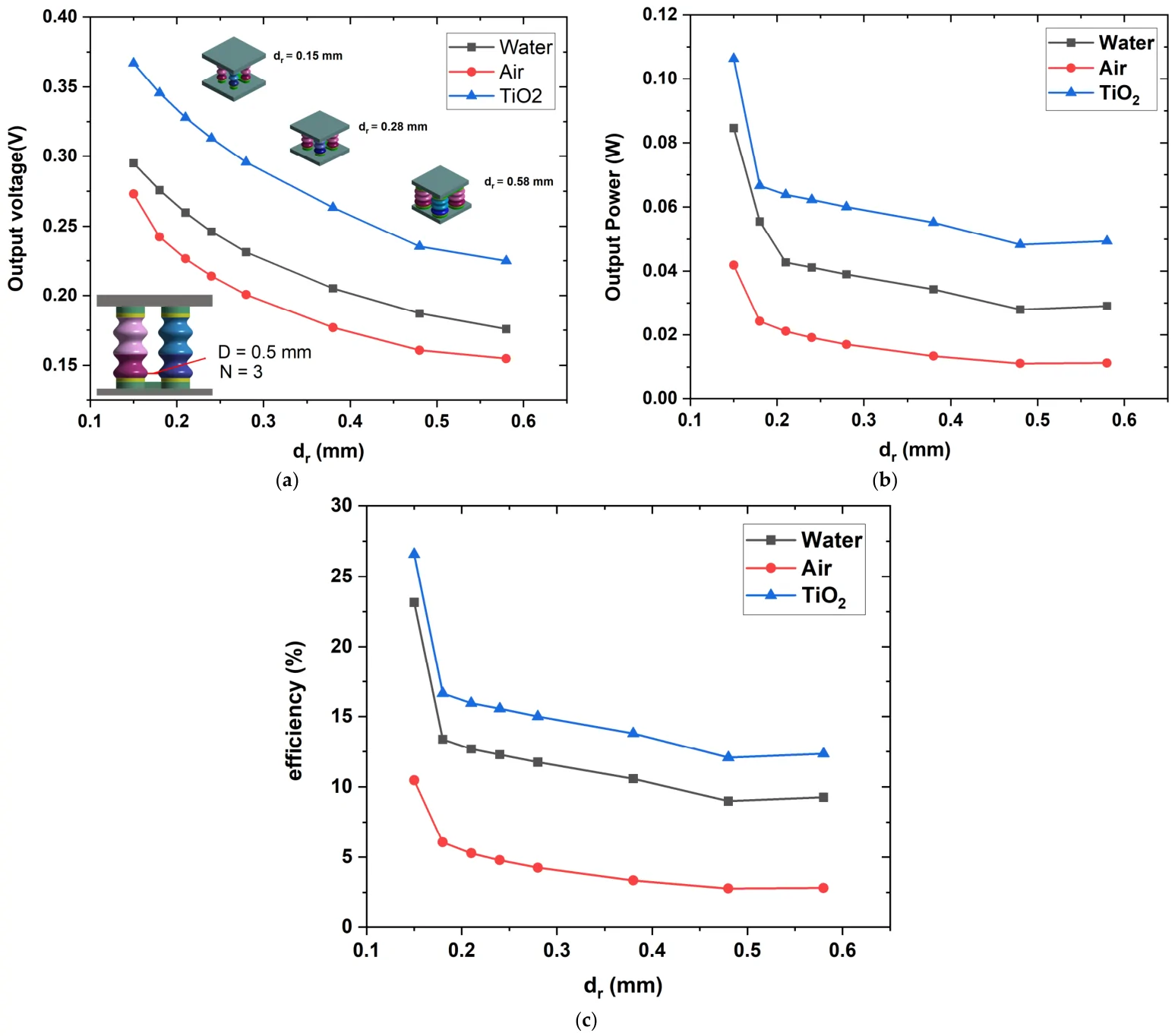
Effect of the revolution distance on the thermoelectric performance of the sinusoidal thermoelectric generator: (**a**) output voltage, (**b**) output power, and (**c**) conversion efficiency, under a constant heat flux of 25,000 W/m and a fixed thermoelement spacing of 0.5 mm. Results correspond to air, water, and nanofluid cooling illustrating the decrease in voltage with increasing $d_{r}$.

**Figure 14:**
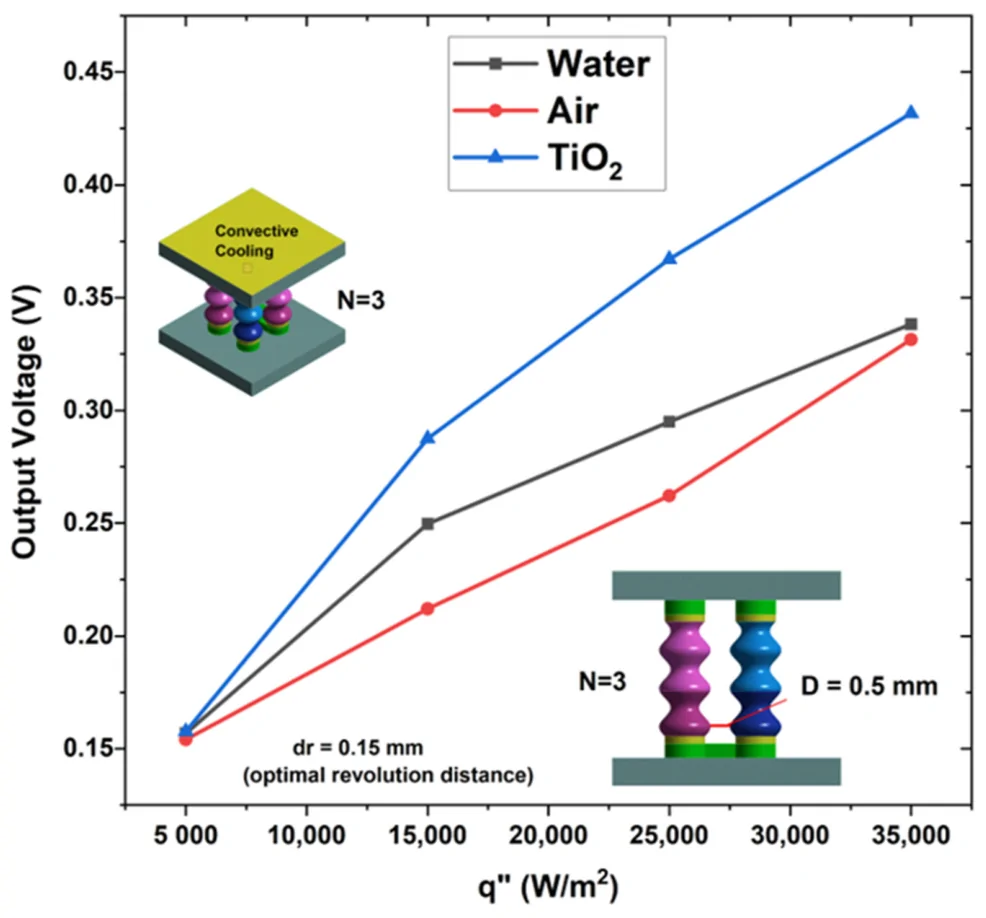
Output voltage versus heat flux for a sinusoidal thermoelectric generator (N=3,dr=0.15 mm) under different convective cooling conditions (air, water, and ${TiO}_{2}$-based nanofluid). Light blue and light pink represent p- and n-type $Bi_{2}Te_{3},$ respectively, while dark blue and dark pink represent p- and n-type skutterudite, respectively.

**Figure 15:**
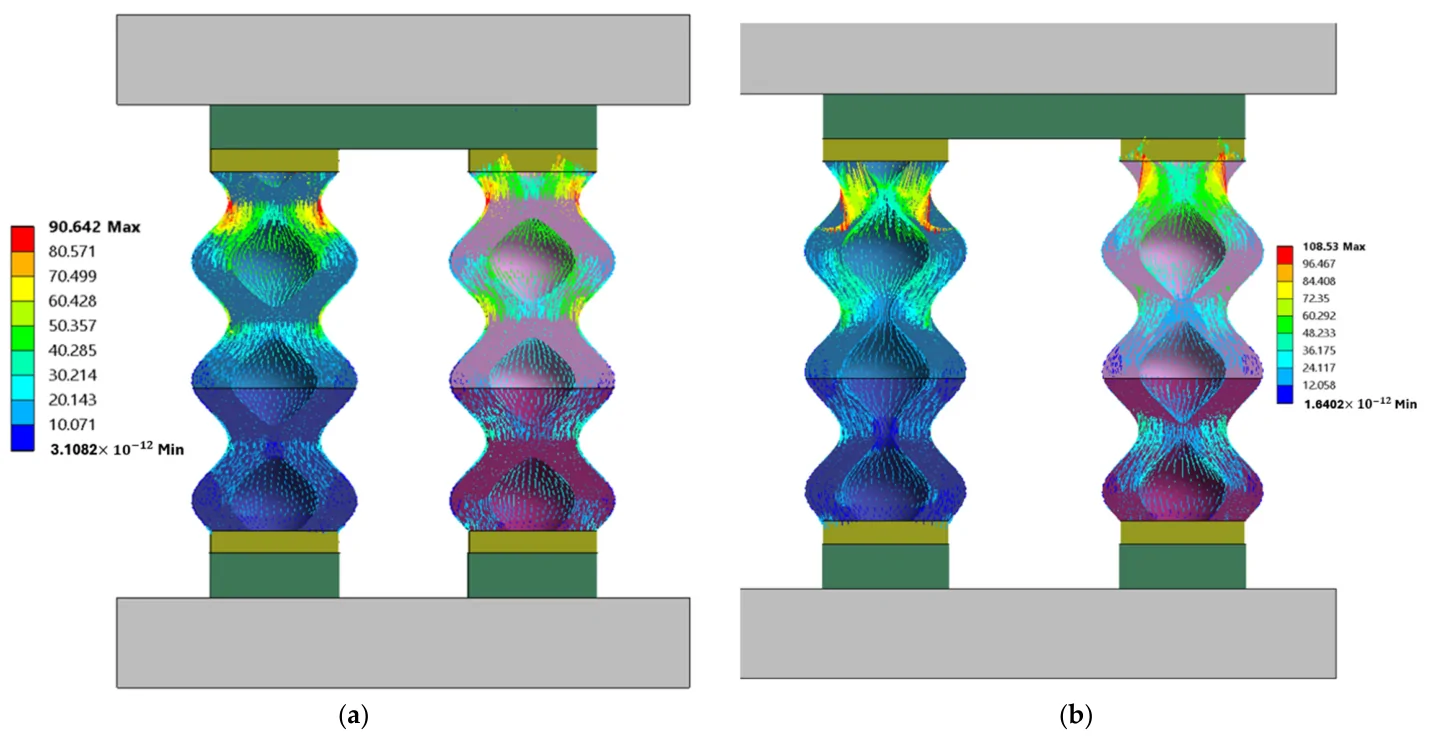
Electric field intensity contours in the thermoelectric legs for two simulated cases: (**a**) D = 0.05 mm (**b**) D = 0.75 mm.

**Figure 16:**
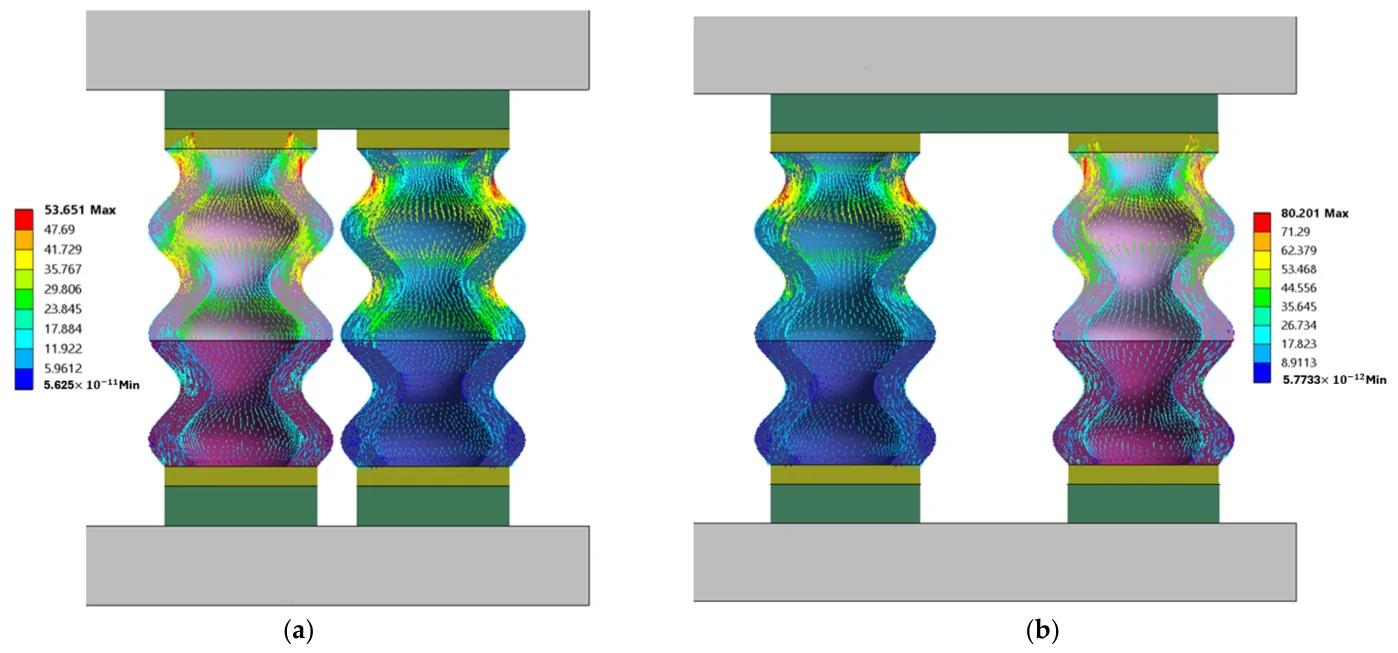
Effect of the separation distance on the electric field intensity distribution within the thermoelectric elements $(d_{r}=0.28\;mm):$ (**a**) D = 0.05 mm and (**b**) D = 0.75 mm.

**Figure 17:**
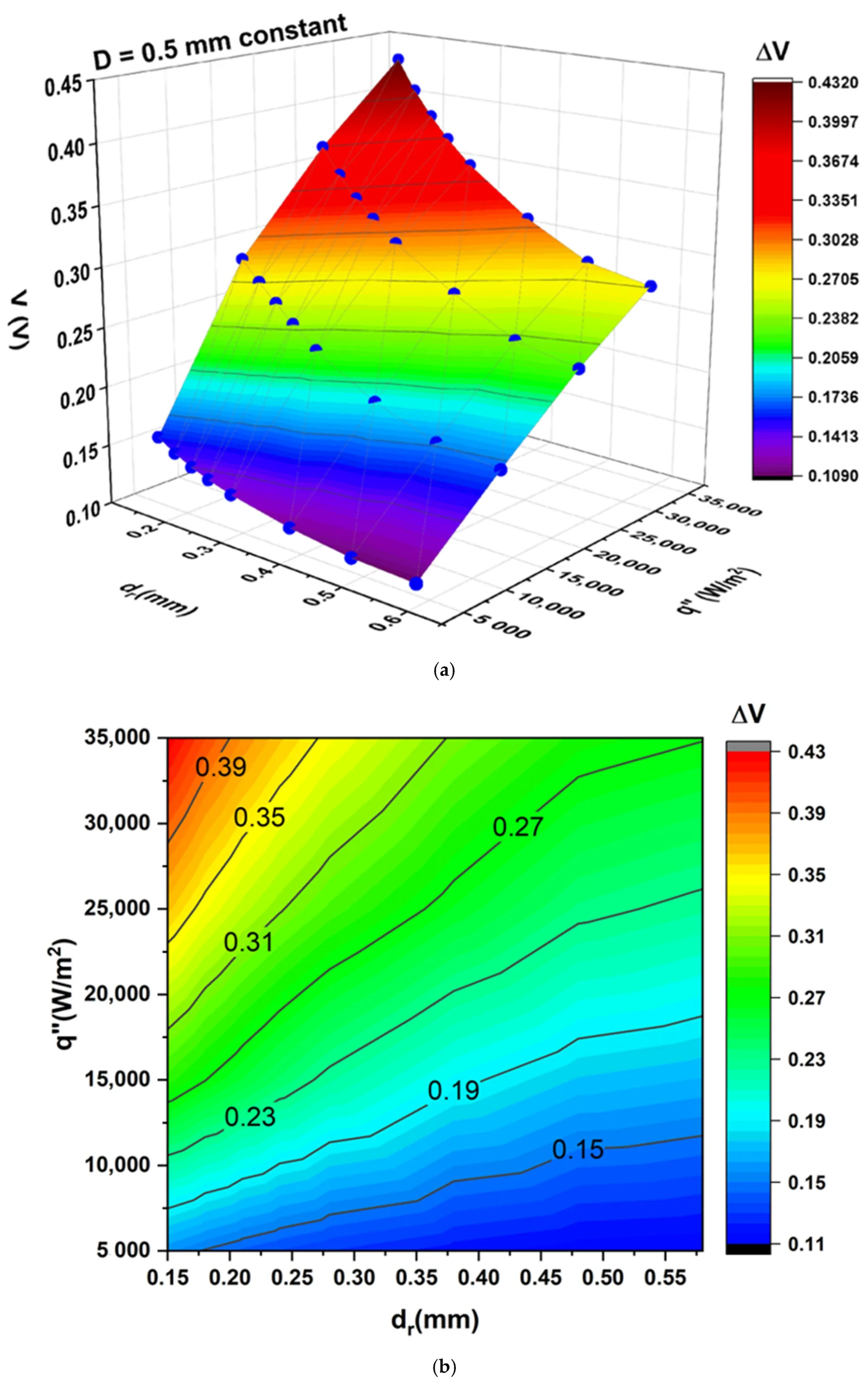
Effect of heat flux (q″) and revolution distance (dᵣ) on the output voltage (ΔV), considering a 0.5 mm inter-leg spacing and convective cooling with TiO_2_ nanofluid: (**a**) response surface and (**b**) contour map.

**Figure 18:**
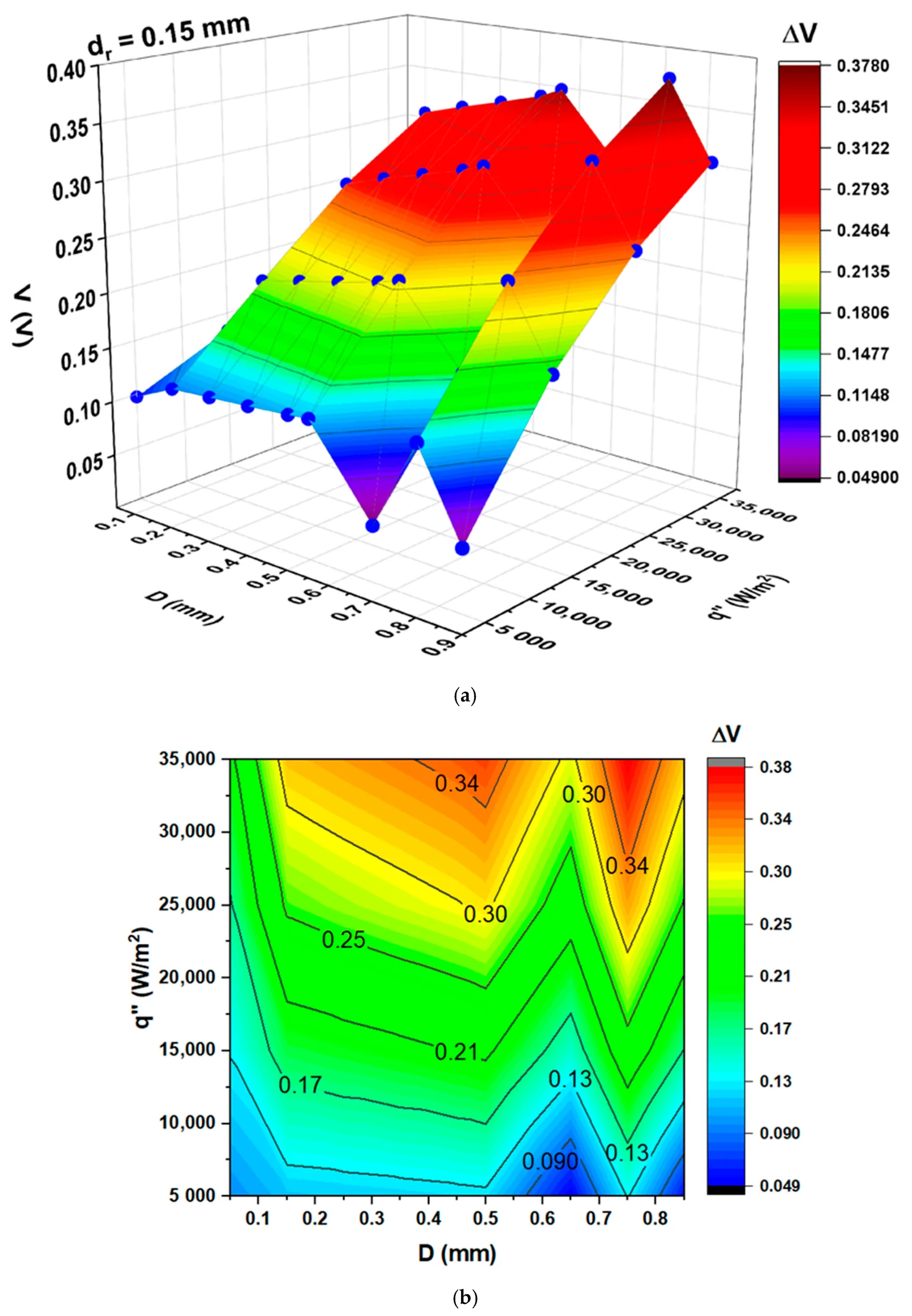
(**a**) Surface plot and (**b**) corresponding contour map of the output voltage (ΔV) as a function of heat flux (q”) and inter-leg spacing (D), with the revolution distance fixed at $d_{r}$ = 0.15 mm.

**Figure 19:**
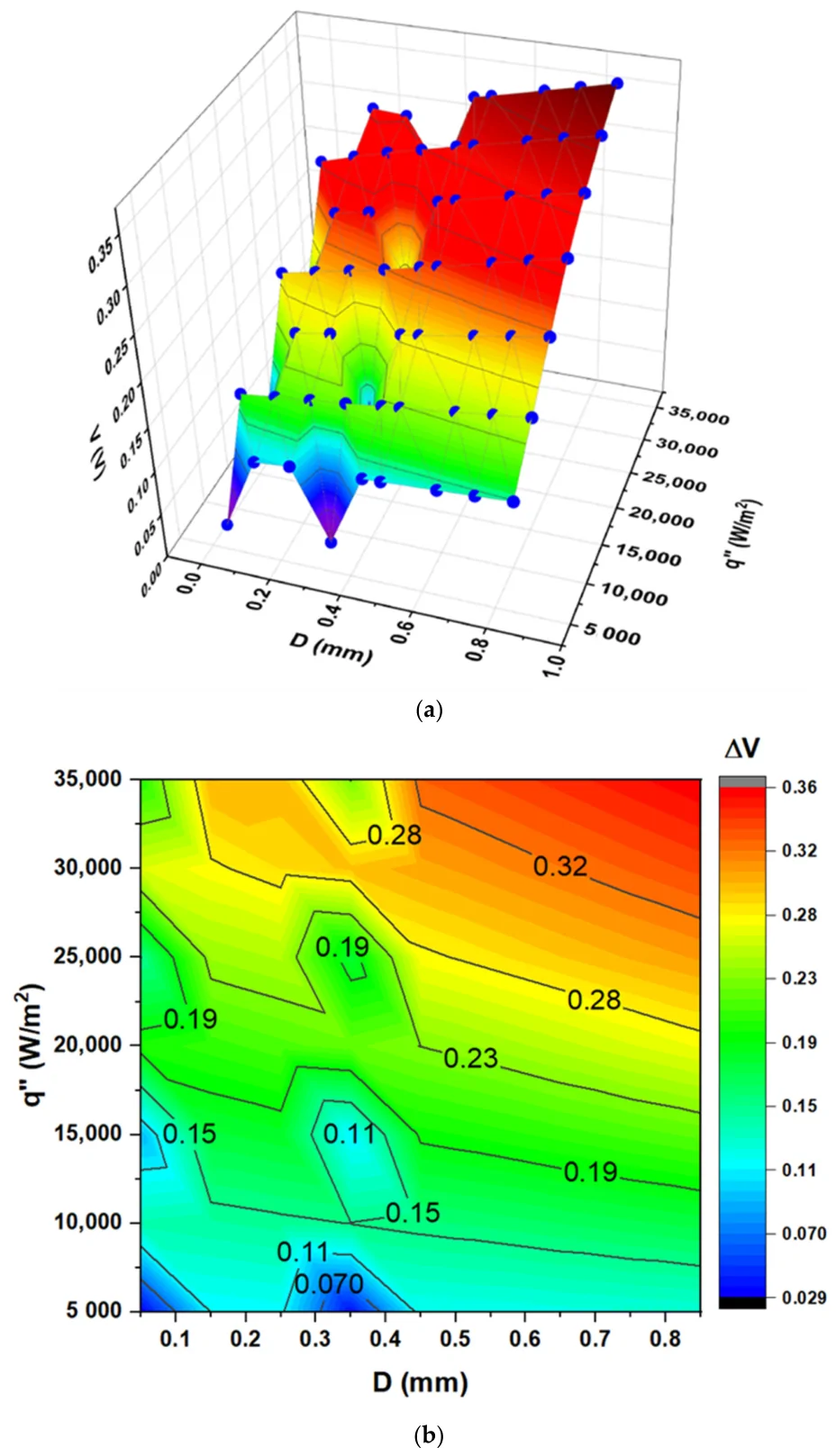
(**a**) Three-dimensional surface and (**b**) contour projection illustrating the variation of the output voltage (ΔV) as a function of heat flux ($q^{\prime \prime }$) and separation distance (D) in Case 2, for a fixed revolution distance ($d_{r}=0.28$ mm).

**Table 1:** Additional material properties included in the numerical simulations [19,20].

Material	Coefficient of Thermal Expansion (1/°C)	Density (kg/$m^{3}$)	Young’s Modulus (GPa)	Heat Capacity (J/kg⋅°C)	Poisson’s Ratio	Electrical Conductivity (S/m)	Thermal Conductivity (W/m⋅°C)
Solder	2.7 × $10^{-5}$	7240	44.5	210	0.33	2 × $10^{7}$	55
Alumina	0.68 × $10^{-5}$	3970	340	800	0.22	1 × $10^{-12}$	25
Copper	1.7 × $10^{-5}$	8930	120	386	0.3	5.9 × $10^{7}$	385
Skutterudites	0.8 × $10^{-5}$	6800	110	225	0.23	-	-
Bismuth Tellurides	0.8–1.32 × $10^{-5}$	7740	65–59	154.4	0.21	-	-

**Table 2:** Mesh-independence study.

Total Number of Mesh Elements	Voltage (V)	Relative Error (%)
4302	0.064779	-
6154	0.064074	1.10
10,828	0.064027	0.073
16,775	0.06401	0.027
51,350	0.064037	0.042
109,704	0.064011	0.041
146,968	0.064042	0.048

**Table 3:** Thermophysical properties of the nanofluids used in the simulations [34].

	$\left[\kappa \right]\;W/m$·°C	$\left[\nu \right]\;10^{-7}m^{2}/s$	$\left[\rho \right]\;kg/m^{3}$	[Cp] J/kg·°C	Concentration (wt%)
Distilled water	0.6	7.477797	997	4181.7	0 wt%
$\mathrm{Ti}O_{2}$	0.607	9.2143	1048	3502	0.1 wt%
$Fe_{3}O_{4}$	0.6274	8.174939	1003.06	4178.485	0.1 wt%
${Al}_{2}O_{3}$	0.661	10.07544	1007.4	4154.7	0.1 wt%
Graphene	0.752	9.97062	1123.3	3343.1888	0.1 wt%

**Table 4:** Convective boundary-condition parameters employed in numerical simulations.

Nanofluid	Reynolds	Velocity (m/s)	Prandtl	Nusselt Number	h (W/$m^{2}$·K)
Distilled water	2500	0.46737	5.19613	43.724	6558.6
Ti$O_{2}$	2500	0.49001	5.57122	44.752	5778.4
F$e_{3}O_{4}$	2500	0.45984	5.46117	42.921	6732.1
${Al}_{2}O_{3}$	2500	0.06721	0.68096	22.209	3670.1
Graphene	2500	0.53023	4.97921	43.107	6895.6

**Table 5:** Comparison of voltage predictions between the FEM results reported by li and Moria and the present model.

Q (W/m^2^)	N	Ali & Moria FEM (V)	Present Study FEM (V)	Difference (%)
5000	1	0.039	0.041597	6.66
5000	2	0.04	0.043133	7.83
5000	3	0.046	0.048938	6.39
5000	4	0.055	0.056225	2.23
15,000	1	0.111	0.11136	0.32
15,000	2	0.122	0.12787	4.81
15,000	3	0.134	0.13482	0.61
15,000	4	0.152	0.1511	0.59
25,000	1	0.176	0.18116	2.93
25,000	2	0.191	0.19163	0.33
25,000	3	0.207	0.20728	0.14
25,000	4	0.227	0.22728	0.12
35,000	1	0.226	0.23345	3.3
35,000	2	0.244	0.24852	1.85
35,000	3	0.265	0.26974	1.79
35,000	4	0.292	0.29206	0.02

**Table 6:** Internal electrical resistance ($R_{int}$) of the TEG for the different revolution distances ($d_{r}$) considered in this study.

$d_{r}$ (mm)	$R_{int}$ (Ω)
0.15	0.19280361
0.18	0.26696032
0.21	0.23911289
0.24	0.21677867
0.28	0.19284543
0.38	0.15135176
0.48	0.1247116
0.58	0.10611065

## Data Availability

The original contributions presented in this study are included in the article. Further inquiries can be directed to the corresponding author.

## Abbreviations

Symbol	Description	Units
$A_{w}$	Wave amplitude of sinusoidal leg	mm
D	Inter-leg spacing distance	mm
$d_{r}$	Revolution distance	mm
$h_{l}$	Thermoelectric leg height	mm
$h_{i}$	Copper interconnector thickness	mm
$h_{s}$	Solder layer thickness	mm
$h_{c}$	Ceramic layer thickness	mm
N	Number of sinusoidal cycles	-
$q^{\prime \prime }$	Applied heat flux	${W/m}^{2}$
h	Convective heat transfer coefficient	${W/m}^{2}$·°C
$T_{h}$	Hot-side temperature	°C
$T_{c}$	Cold-side temperature	°C
ΔT	Temperature difference	°C
ΔV	Generated voltage	V
I	Electric current	A
J	Electric current density	A/$m^{2}$
Nu	Nusselt number	-
Re	Reynolds number	-
Pr	Prandtl number	-
κ	Thermal conductivity	W/$m^{2}$· °C
α	Seebeck coefficient	V/°C
ϕ	Electric potential	V
ρ	Density	kg·$m^{3}$
$C_{p}$	Specific heat capacity	J/kg·°C
ν	Kinematic viscosity	$m^{2}$/s
Abbreviation	Description	
TEG	Thermoelectric Generator	
STEG	Segmented Thermoelectric Generator	
FEM	Finite Element Method	
CFD	Computational Fluid Dynamics	
TE	Thermoelectric	
ZT	Thermoelectric Figure of Merit	
$Bi_{2}Te_{3}$	Bismuth Telluride	
$TiO_{2}$	Titanium Dioxide	
$Al_{2}O_{3}$	Aluminum Oxide	
$Fe_{3}O_{4}$	Magnetite	
